# Identification and validation of parthanatos-related genes in end-stage renal disease

**DOI:** 10.1080/0886022X.2025.2519834

**Published:** 2025-07-06

**Authors:** Xuan Dai, Lianfang Yuan

**Affiliations:** a Tianjin Key Laboratory of Acute Abdomen Disease Associated Organ Injury and ITCWM Repair, Institute of Integrative Medicine for Acute Abdominal Diseases, Tianjin NanKai Hospital, Tianjin Medical University, Tianjin, China; b NHC Key Lab of Hormones and Development and Tianjin Key Lab of Metabolic Diseases, Tianjin Medical University Chu Hsien-I Memorial Hospital & Institute of Endocrinology, Tianjin, China

**Keywords:** End stage renal disease, parthanatos, immune regulation, machine learning, diagnostic

## Abstract

**Background:**

End-Stage Renal Disease (ESRD) is a severe chronic kidney disease with a rising global incidence, often accompanied by various complications, severely impacting patients’ quality of life. Parthanatos plays a crucial role in the pathogenesis of multiple diseases. This study aims to explore the role of parthanatos-related genes in ESRD through bioinformatics analysis.

**Methods:**

In this study, blood samples from ESRD patients and healthy controls were analyzed using public transcriptomic data. Two machine learning algorithms identified candidate genes, refined through ROC analysis. A nomogram assessed their predictive potential for ESRD prevalence. Gene Set Enrichment Analysis (GSEA) and immune infiltration analysis confirmed their roles in immune functions. The relationship between the two identified biomarkers and ESRD was investigated through molecular and disease networks, enhancing understanding of their association. Clinical validation of biomarker expression was conducted using reverse transcription-quantitative polymerase chain reaction (RT-qPCR).

**Results:**

The 65 candidate genes were refined by PPI network, screened by two algorithms, and then determined by ROC analysis to obtain HBM and MYL4 as biomarkers. The nomogram constructed for these two biomarkers demonstrated their effectiveness in predicting survival outcomes among ESRD patients. Notably, there is a strong correlation between HBM and MYL4 with Type 17 T helper cells and central memory CD4 T cells RT-qPCR validation showed that the expression of biomarkers in ESRD patients was significantly higher than that in controls (*p* < 0.05).

**Conclusion:**

This study identified two biomarkers (HBM, MYL4) through transcriptome analysis, investigating their functions and mechanisms, offering new therapeutic insights for ESRD.

## 1. Introduction

End-Stage Renal Disease (ESRD) is a clinical syndrome caused by a series of symptoms and metabolic disorders caused by various kidney diseases [1]. Common causes of ESRD include diabetic nephropathy, chronic glomerulonephritis, hypertensive nephropathy, congenital kidney diseases, and various autoimmune kidney diseases. Ultimately, the primary diseases cause structural damage to the glomeruli or a decline in the glomerular filtration rate, leading to the inability to effectively filter metabolic waste from the circulating blood, which is the direct cause of the resulting pathophysiological changes [2]. ESRD has an incidence rate as high as 1 in 10,000 and can lead to multiple clinical complications. ESRD patients often suffer from severe complications such as hypertension, coronary artery disease, hyperkalemia, heart failure, as well as nausea, skin itching, anemia, seizures, and even coma [3]. These conditions affect multiple body systems and major organs and can also lead to psychological issues like depression and anxiety, significantly impacting the patients’ quality of life and causing varying degrees of physical and psychological distress. The primary treatment for ESRD is renal replacement therapy, including kidney transplantation, hemodialysis, and peritoneal dialysis. However, due to the high cost of organ transplantation and donor shortages, hemodialysis (HD), automated peritoneal dialysis (APD), and continuous ambulatory peritoneal dialysis (CAPD) are the main treatment options for ESRD [4]. Given the high incidence and multiple complications, the treatment of ESRD is costly, and both hospitalization and mortality rates are high. ESRD has become a major public health issue for the Chinese government. Therefore, early diagnosis and treatment of ESRD are critically important.

Parthanatos is a form of programmed cell death related to increased PARP activity, with its molecular mechanism primarily involving PARP1-mediated apoptosis-inducing factor (AIF) and macrophage migration inhibitory factor (MIF)-dependent programmed cell death [5,6]. As a unique form of cell death, Parthanatos holds a distinct position in cell death research. Its key distinguishing feature from apoptosis is its dependence on the overactivation of PARP-1, which leads to extensive ADP-ribosylation and subsequent cellular energy depletion, playing a crucial role in the pathogenesis of several diseases [6,7]. Research on Parthanatos has made progress, and its pathophysiological role is being explored in neurodegenerative diseases such as Alzheimer’s disease, tumor progression, autoimmune diseases, cardiovascular diseases, and renal diseases [8–11]. Both excessive and defective parthanatos can lead to pathological cell damage, which is crucial for the treatment and prevention of diseases. However, no studies have explored the mechanism of parthanatos in ESRD. Therefore, investigating the mechanism of PRGs in ESRD holds significant importance for the prevention and treatment of this condition.

Therefore, this study utilized transcriptomic data of ESRD from public databases and employed bioinformatics techniques to explore the diagnostic potential of PRGs as biomarkers for ESRD and its potential molecular regulatory mechanisms, providing new references for the clinical diagnosis, prevention, and treatment of ESRD.

## 2. Methods

### 2.1. Data collection

The ESRD dataset was downloaded from Gene Expression Omnibus (GEO, https://www.ncbi.nlmnih.gov/geo/). Downloaded microarray dataset GSE37171 (63 ESRD blood samples and 20 normal blood samples, sequencing platform GPL570) and GSE142153 (7 ESRD blood samples and 10 normal blood samples, sequencing platform GPL6480) as the training and validation sets [12], respectively. Besides there were 11 parthanatos-related genes (PRGs), including PARP1, AIFM1, ADPRHL2, RNF146, NAMPT, GPX4, MAPK8, SQSTM1, CAST, AIMP2, and RIPK1 [13]. The flowchart of this study was shown in Supplementary Figure 1.

### 2.2. Differential expression analysis

The differentially expressed genes (DEGs) between ESRD and normal samples in GSE37171 dataset were identified using the ‘Limma’ R package (v 3.54.0) [14]. Using |log2Fold Change| > 1 and p.adj < 0.05 as the screening criteria. The R package ‘ggplot2’ (v 3.4.1) [15] was used to draw the volcano map of the DEGs, and the R package ‘pheatmap’ (v 1.0.12) (https://CRAN.R-project.org/package=pheatmap) was used to visualize the heatmap.

### 2.3. Weighted gene co-expression network analysis (WGCNA)

The aim was to identify modular genes linked to parthanatos in ESRD. Initially, the ssGSEA algorithm in the ‘GSVA’ package (1.42.0) [16] was employed to measure the PRGs scores for the dataset’s samples, followed by comparison of PRGs scores of ESRD and controls, followed by a comparison with the control group, employing the Wilcoxon rank sum test to assess score disparities between the ESRD and control groups, setting a screening criterion of *p* < 0.05.

Then, the PRGs score was used as a phenotype in this study, co-expression network was constructed using the R package ‘WGCNA’ (v 1.70.3) [17]. Firstly, using the ‘GoodSamplesGenes’ function hierarchical clusters all the samples in the GSE37171 were made to identify and exclude outliers. Subsequently, R^2^ = 0.8 was set and average connectivity was set to 0 to screen for a soft threshold above the red cut line (β). Gene adjacency was then calculated, which led to computing gene similarity, from which a gene dissimilarity coefficient was derived to create a hierarchical clustering tree of genes. Set the minimum gene count per module to 100 and the module merge parameter to 0.25. Finally, in order to identify the key module genes, the genes within the module were analyzed and those with Module Membership (MM) > 0.4 and Gene Significance (GS) > 0.4 were selected as the key module genes.

### 2.4. Identification and analysis of candidate genes

In order to obtain candidate genes, the ‘VennDiagram’ R package (v 1.7.3) [18]was used to intersect the DEGs with the key module genes, the overlapping gene were labeled as candidate genes. In this study, the Gene Ontology (GO) and Kyoto Encyclopedia of Genes and Genomes (KEGG) pathway enrichment of the candidate genes were analyzed using the R package ‘clusterProfiler’ [19] to search for the common functions of the candidate genes and the related pathways, according to the standard of p.adj < 0.05. Additionally, candidate genes were analyzed by protein-protein interaction (PPI) networks using data from the STRING database (https://string-db.org/) (high confidence >0.4), and the results were visualized using Cytoscape [20].

### 2.5. Machine learning

Key genes were screened using a machine learning approach based on all samples in GSE37171. Firstly, the candidate genes were analyzed by XGBoost using the R package ‘XGBoost’ (v 1.7.3.1) [21], with learning rate parameter eta = 0.3 and depth parameter max_depth = 2 for each decision tree. Subsequently, candidate genes were analyzed by Boruta using the ‘Boruta’ package [22], setting the parameters of the algorithm were set to pValue = 0.01. And further, defined the intersecting genes of two machine learning screens as key genes and using the R package ‘VennDiagram’ (v 1.7.3) [18] to draw a Venn diagram.

### 2.6. ROC analysis

Based on GSE37171, the R package ‘pROC’ (v 1.18.5) [23] was utilized to conduct receiver operating characteristic (ROC) analysis of the key genes, calculate area under the ROC curve (AUC), and retain those key genes with AUC > 0.7. The same procedure was carried out for the validation set. The key genes that met the criteria in both datasets were defined as candidate biomarkers. To determine the expression changes between ESRD disease samples and normal samples, analysis was conducted on the manifestation of potential biomarkers within the training and validation datasets. Genes with significant differences and consistent trends were selected for the comparison of expression using ‘rstatix’ and Wilcoxon’s rank-sum test (*p* < 0.05)

### 2.7. Construction of nomogram

To investigate whether biomarkers predicted the prevalence of ESRD, the nomogram based on biomarkers was constructed using the ‘rms’ package [24] in all samples of the GSE37171. The calibration curve is then plotted using the ‘rms’ package. In order to further evaluate the reliability of the diagnosis of the disease, the corresponding ROC curves were plotted with the help of the R package ‘pROC’ (1.18.0) [23], and the predictive value of the diagnosis of the disease was evaluated by the AUC value of the nomogram model.

### 2.8. Gene set enrichment analysis

For elucidating the signaling routes linked to the biomarkers, refer to the c2.cp.kegg.v7.4.symbols.gmt genomic dataset obtained from the MSigDB database. Spearman correlation analyses were performed for each biomarker and other genes in the training set using the R package ‘psych’ (v 2.1.6) [25], and the correlation coefficient was used as the ranking criterion. The GSEA function within the R package ‘clusterProfiler’ was employed to analyze and visualize biomarkers in every sample of the training set. Threshold *p* < 0.05, enrichment significant at |NES|>1.

### 2.9. Immune infiltration analysis

To assess the prevalence of 28 immune cells [26] in every GSE37171 sample, an analysis of these cells’ infiltration was conducted using the ssGSEA algorithm and the ‘GSVA’ R package (1.42.0) [16] and the distribution ratio of 28 immune cell types in the samples was observed by plotting stacked plots using the R package ‘ggplot2 R package. Moreover, the R package ‘rstatix’ (https://CRAN.R-project.org/package=rstatix) was used within GSE37171 database to screen out the immune cells with significant difference (*p* < 0.05) between disease and normal samples by Wilcoxon test as differential immune cells. Subsequently, Spearman analyses using the ‘psych’ R package were conducted to investigate the relationship between differentiated immune cells in ESRD and biomarkers and differentiated immune cells, and all samples in the training set GSE37171 were analyzed separately for significantly differentiated immune cells and biomarkers and significantly differentiated immune cells were analyzed.

### 2.10. Establishment of molecular regulatory networks

The target miRNAs of the biomarkers were predicted using the Microcosm database (https://mycocosm.jgi.doe.gov/mycocosm/home), the miRNA-related lncRNAs were predicted with the help of the starbase database (https://ngdc.cncb.ac.cn/databasecommons/database/id/169), and the results were subsequently visualized using the R package ‘MultiMiR’ (v 3.19) [27]. ‘MultiMiR’ was used to visualize the results to construct lncRNA-miRNA-mRNA (biomarker) networks.

### 2.11. Disease association analysis

Searched for other diseases that were associated with biomarkers and might share the same molecular mechanisms as ESRD to explore potential approaches and effective strategies for the treatment of ESRD. The biomarkers were entered into DisGeNet (https://www.disgenet.org/) to screen for biomarker-associated diseases. The biomarker-disease association networks were built for visualization using NetworkAnalyst (https://www.networkanalyst.ca/) and Cytoscape software [20].

### 2.12. RT-qPCR validation

Experimental samples including 5 ESRD samples and 5 control samples were gained from Tianjin NanKai Hospital, Tianjin Medical University. All samples underwent reverse transcription-quantitative polymerase chain reaction (RT-qPCR). This study received approval from Ethics Committee of Nankai Hospital. To validate the expression of biomarkers, total RNA was extracted from the samples using TRIZOL (Ambion, Austin, USA) following the manufacturer’s instructions. The first strand of the complementary DNA (cDNA) was synthesized from 2 μg of total RNA using the SweScript First Strand cDNA Synthesis Kit (Servicebio, Wuhan, China) according to the provided, guidelines. RT-qPCR was conducted using the 2xUniversal Blue SYBR Green qPCR Master Mix (Servicebio, Wuhan, China). The reaction program was set as follows: 1 min at 95 °C, 40 cycles at 95 °C for 20 s and 55 °C for 20 s, and 30 s at 72 °C. Primer sequenes were listed in Table S1. GAPDH was used as an internal reference gene. Gene expression levels were calculated using the 2^-△△Ct^ method [28].

### 2.13. Statistical analysis

R software version 4.2.3 was utilized to process and analyze the data. Group comparisons were performed using the Wilcoxon test. The determination of statistical relevance was based on a p-value below 0.05.

## 3. Results

### 3.1. A Total of 65 candidate genes were screened

**Figure 1.**
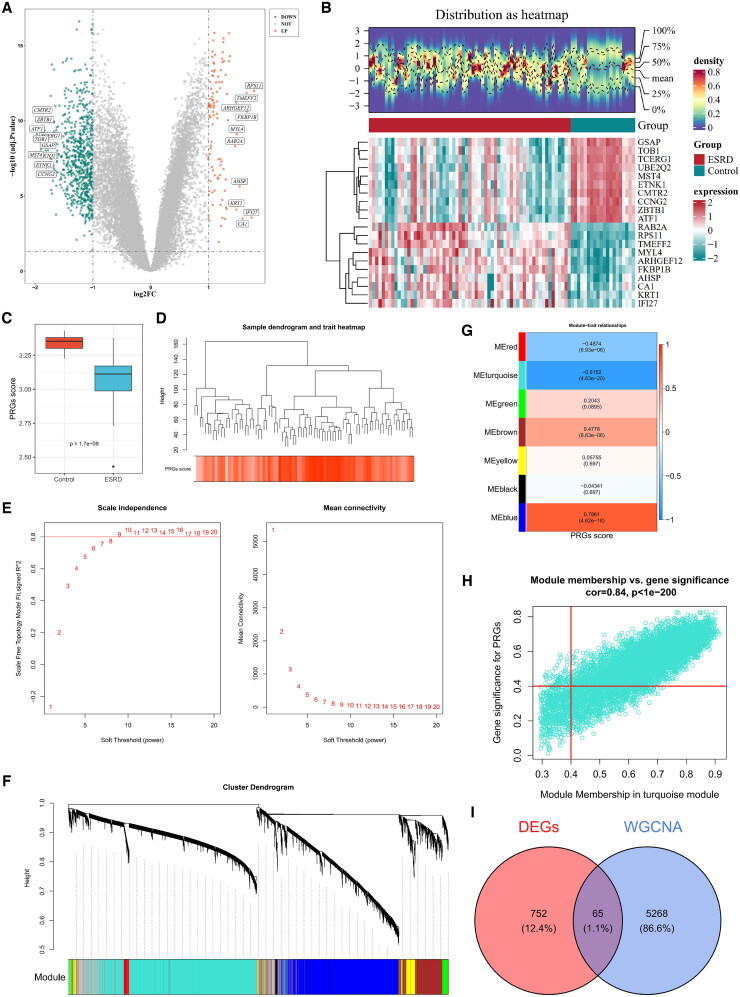
Identification of candidate genes. **(A)** Volcano plot of differentially expressed genes. The y-axis represents -log10(adj.p.value), and the x-axis represents the fold change log2FC. Each dot represents a gene; orange dots indicate upregulated genes, green dots indicate downregulated genes, and gray dots indicate genes with no significant expression. The top 10 most significantly upregulated and downregulated genes are labeled with their names. **(B)** Heatmap of differentially expressed genes. The figure has two parts. The upper part shows the density heatmap of up regulated and down regulated gene expression levels across samples, displaying lines for five quantiles and the mean. The lower part represents a heatmap. It displays the top 10 downregulated and top 10 upregulated genes based on log2FC ranking, showing gene expression levels across different samples. The heatmap color indicates gene expression levels, with green representing the control group and red representing the ESRD group. **(C)** Differences in ssGSEA scores of PRGs between ESRD and normal control groups. The x-axis represents the different groups, and the y-axis represents the ssGSEA scores of PRGs. The line in the Middle of the box indicates the median, representing the average level of sample data. **(D)** Hierarchical clustering analysis. Each branch in the clustering tree represents a sample, and the y-axis indicates the euclidean distance of gene expression levels among samples. **(E)** Selection of soft thresholds. **(F)** Identification of co-expression module. The figure consists of two parts: the upper part shows a hierarchical clustering dendrogram of genes, and the lower part depicts gene modules. **(G)** Heatmap of correlation between modules and ssGSEA scores. The color blocks on the left represent modules, and the color bar on the right represents the correlation range. In the heatmap, deeper colors indicate higher correlations, with red showing positive correlations and blue showing negative correlations. The numbers in each cell represent correlation coefficients and significance levels. **(H)** Identification of key module genes. The x-axis represents the correlation between each gene in the selected module and the module itself, while the y-axis represents the correlation between the gene and the trait. **(I)** Venn diagram of candidate genes.

The analysis results revealed 817 DEGs between the ESRD and Control groups in the GSE37171 database, with 99 up-regulated genes and 718 down-regulated genes (Figure 1(A)). Furthermore, the top 10 most significantly up-regulated and down-regulated genes from the plot were selected and presented in the heatmap to showcase their expression levels (Figure 1(B)).

There was a notable disparity (*p* < 0.05) in PRGs scores between the groups with ESRD and the control group (Figure 1(C)). This finding was important for elucidating the molecular mechanisms of cell death and identifying potential therapeutic targets for ESRD. Hierarchical cluster analysis of ESRD samples in the training set revealed no outliers (Figure 1(D)). Subsequently, when the β-value was set to 9, the R^2^ =0.8 (red line) and the mean connectivity converged to 0, at which point the network was most consistent with a scale-free network distribution state and biologically significant (Figure 1(E)). Then, the WGCNA analysis identified a total of 2 key modules (excluding the grey module, which comprises genes that could not be categorized into any specific module) (Figure 1(F)). Then, 7 co-expression modules were identified and the turquoise module (|cor| = 0.82, *p* < 0.05), which had the highest correlation with ESRD scores, was selected as the key module, and a total of 7, 050 genes were included in the module (Figure 1(G)). Genes with |MM|>0.4 and |GS|>0.4 were selected as key modular genes, resulting in 5,333 genes for subsequent analysis (Figure 1(H)). Finally, the 817 differentially expressed genes obtained in the above study were cross-analyzed with 5,333 key module genes, resulting in 65 candidate genes (Figure 1(I)).

### 3.2. Candidate genes enrichment analysis and PPI network construction

**Figure 2.**
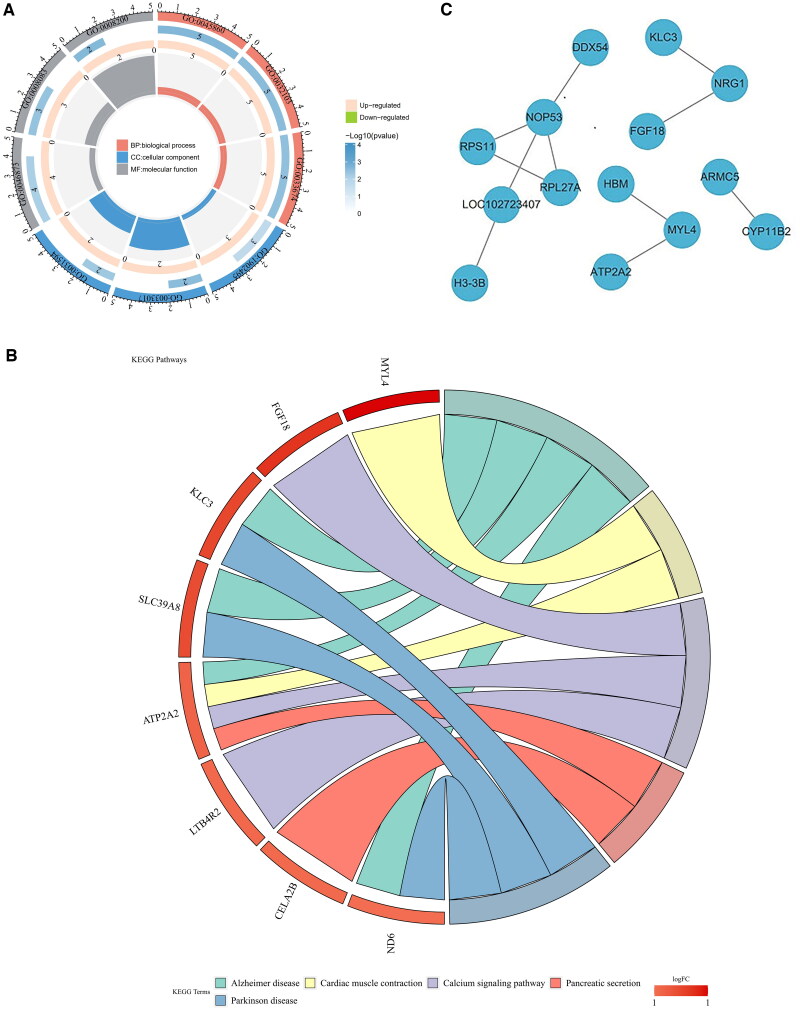
Enrichment analysis of candidate genes. **(A)** GO enrichment analysis of candidate genes. The outermost layer of the chart represents GO annotation function IDs, with red indicating biological processes, blue indicating cellular components, and gray indicating molecular functions. The second layer’s color depth indicates significance levels, and the size and numbers represent the number of genes enriched in the function. The third layer depicts the number of upregulated and downregulated genes enriched in the function, with color indicating gene regulation. The innermost layer’s color blocks represent different functions, with size indicating the RichFactor. **(B)** KEGG enrichment analysis of candidate genes. The circular chart has two halves. The left half shows enriched gene names, with color depth representing logFC values (deeper color indicates greater fold change). The right half represents enriched pathways, with different colors corresponding to different pathways. The size of the color blocks changes with the number of enriched genes; larger blocks indicate more genes in the pathway. Lines in the Middle connect genes enriched in different pathways. **(C)** PPI network of candidate genes. Nodes represent genes, and edges represent interactions between genes.

For the 65 intersecting genes, GO functional annotation yielded a total of 317 enriched results, which were categorized into 259 BP (Biological Processes), 15 CC (Cellular Components), and 43 MF (Molecular Functions) (Figure 2(A)). Notably, significant enrichments were identified in pathways related to positive regulation of protein kinase activity, positive regulation of response to external stimulus, neuromuscular junction, ion channel inhibitor activity, and metal ion transmembrane transporter activity et al. On the other hands KEGG functional enrichment analysis was performed on the 65 candidate genes, resulting in the identification of 5 relevant pathways (Figure 2(B)). The enrichment results were presented visually, revealing that the candidate genes exhibited significant associations with Alzheimer’s disease, Cardiac muscle contraction, Calcium signaling pathway, Pancreatic secretion, and Parkinson’s disease, as evident from the KEGG enrichment outcomes. In addition, PPI network analysis of 65 differentially expressed genes was performed in the STRING database. As illustrated, the network encompasses 14 nodes and 11 edges, there was an interaction between these 14 genes (Figure 2(C)). So, these 14 genes were designated as the final set of candidate genes for further analysis (ARMC5, ATP2A2, DDX54, FGF18, H3-3B, HBM, KLC3, LOC102723407, NOP53, RPL27A, CYP11B2, MYL4, NRG1, and RPS11).

### 3.3. 8 Key genes screened using machine learning

**Figure 3.**
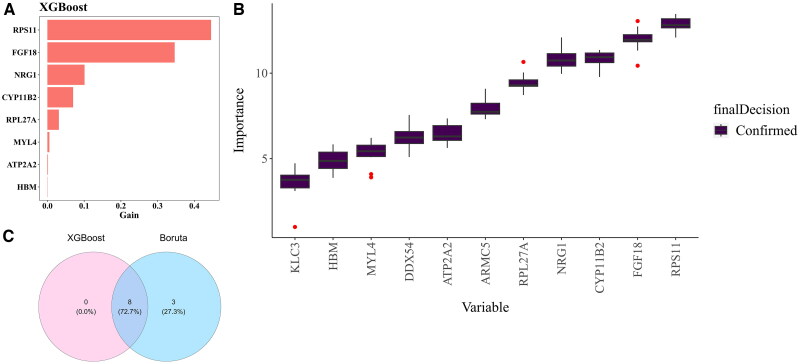
Identification of key genes. **(A-B)** XGBoost machine learning plot. **(C)** Intersection of genes identified by two algorithms to obtain key genes.

Eight candidate genes (RPS11, FGF18, NRG1, CYP11B2, RPL27A, MYL4, ATP2A2, and HBM) were finally identified by XGBoost (Figure 3(A)). In this study, 11 candidate genes have been successfully screened through the Boruta algorithm, including KLC3, HBM, MYL4, DDX54, ATP2A2, ARMC5, RPL27A, NRG1, CYP11B2, FGF18, and RPS11 (Figure 3(B)). The eight intersecting genes of the two algorithms were taken as key genes (RPS11, FGF18, NRG1, CYP11B2, RPL27A, MYL4, ATP2A2, and HBM) (Figure 3(C)).

### 3.4. Critical role and predictive value of HBM and MYL4 in ESRD

**Figure 4.**
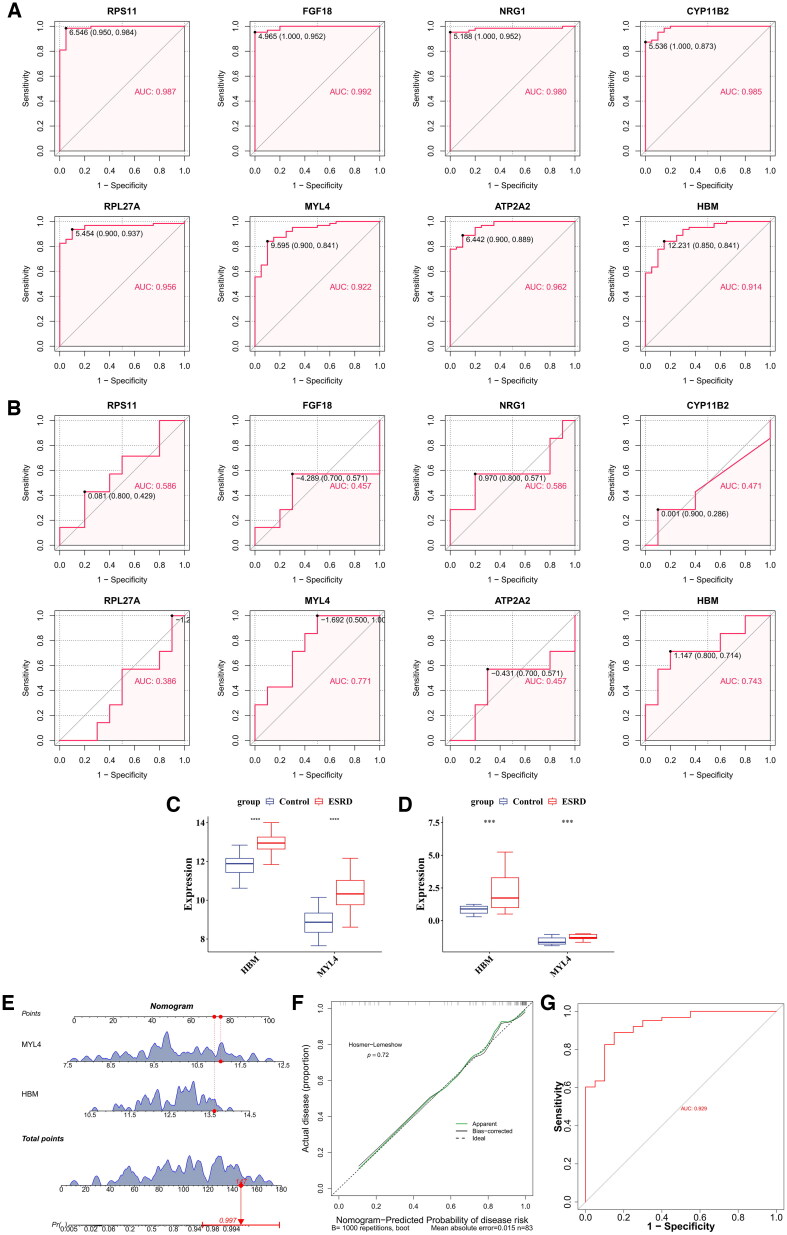
Construction of nomogram model. (A) ROC curve for key genes in training set GSE37171. The x-axis represents specificity, the y-axis represents sensitivity, and the area under the curve (AUC) measures diagnostic performance. The closer the AUC value is to 1, the better the diagnostic performance. (B) ROC curve for key genes in validation set GSE142153. (C) Expression analysis of biomarkers in the training set. (D) Expression analysis of biomarkers in the validation set. (E) Nomogram model based on biomarkers. (F) Calibration curve of the nomogram model. (G) ROC curve of the nomogram model.

The result of the ROC curve for the training set showed that the AUC of the 8 key genes, were all greater than 0.7 (Figure 4(A)). Concurrently, the validation set’s ROC curve analysis revealed that the ROC AUC for the two crucial genes, MYL4 and HBM, exceeded 0.7 (Figure 4(B)). The key genes HBM and MYL4, common to both datasets, were identified as candidate biomarkers. These two candidate biomarkers differed significantly between the training and validation sets and were expressed in disease samples at higher levels than normal samples, and were therefore used as biomarkers (Figure 4(C,D)). A nomogram was developed for every sample in the GSE37171 training set, aggregating individual scores derived from HBM and MYL4 expression levels, culminating in a cumulative score of 147, which corresponds to a probability of disease onset of 0.997 (Figure 4(E)). To assess the predictive performance of the nomogram model, calibration curves were plotted. In this study, the slope of our calibration curve approached 1, with a p-value of 0.72, this underscores the satisfactory predictive accuracy of the nomogram (Figure 4(F)). Furthermore, the results revealed the AUC value of 0.929 for the ROC curve of the nomogram, signifying a robust predictive capability of the nomogram model in diagnosing the disease (Figure 4(G)).

### 3.5. Gene pathways and immune cells in ESRD

**Figure 5.**
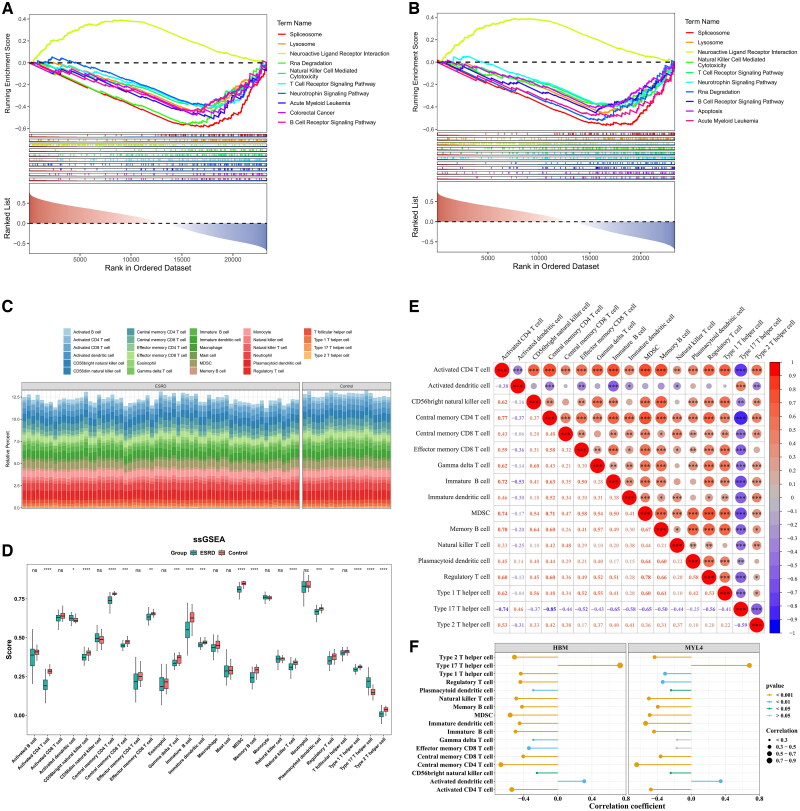
Immune infiltration analysis. **(A)** GSEA enrichment analysis of HBM. **(B)** GSEA enrichment analysis of MYL4. **(C)** Stacked chart of immune cell infiltration in ESRD and control groups. **(D)** Differential expression of immune cells between ESRD and control groups in training set GSE37171. * indicates *p* < 0.05, ** indicates *p* < 0.01, *** indicates *p* < 0.001, and **** indicates *p* < 0.0001. **(E)** Correlation and significance of differential immune cells. Red indicates positive correlation, while blue indicates negative correlation. Circles with * indicate *p* < 0.05, ** indicates *p* < 0.01, *** indicates *p* < 0.001, and **** indicates *p* < 0.0001. Larger circles represent higher significance. **(F)** Correlation between biomarkers and differential immune cells. Circle size represents correlation strength. Green lines indicate *p* < 0.05, blue lines indicate *p* < 0.01, and brown lines indicate *p* < 0.001. The x-axis shows correlation coefficients. The y-axis lists different immune cells.

In the study, GSEA was used for genome-wide enrichment analysis and it was found that the HBM gene was mainly enriched in the pathway such as Neuroactive Ligand Receptor Interaction, Natural Killer Cell Mediated Cytotoxicity, and T Cell Receptor Signaling Pathway. On the other hand, MYL4 genes were predominantly enriched in pathways including Natural Killer Cell Mediated Cytotoxicity, T Cell Receptor Signaling Pathway, and Apoptosis (Figure 5(A,B)).

The stacked plot results revealed distinct proportions of immune cells between ESRD samples and Control samples (Figure 5(C)). There were 17 cell types exhibit significant differences in infiltration between the ESRD and Control groups within the GSE37171 dataset. Specifically, the scores for Activated dendritic cells and Type 17 T helper cells are higher in the ESRD group compared to the Control group (Figure 5(D)). To explore the correlations among the differentially expressed immune cells in ESRD, a correlation analysis was performed on the 17 significantly different immune cell types across all samples in the GSE37171 dataset. As illustrated, the strongest positive correlation was observed between central memory CD4 T cells and activated CD4 T cells (*r* = 0.774, *p* < 0.001), while the strongest negative correlation was detected between central memory CD4 T cells and Type 17 T helper cells (r= −0.846, *p* < 0.001) (Figure 5(E)).

For investigating the correlations between the biomarkers HBM and MYL4 with the differentially expressed immune cells, Spearman correlation analyses were performed in the GSE37171 dataset. According to the lollipop plot, both HBM and MYL4 exhibited high correlations with most immune cell types. HBM and MYL4 showed high correlation with most of the immune cells. Among them, the two biomarkers showed the most significant positive correlation with type 17 T helper cells: the correlation between HBM and type 17 T helper cells was 0.731077621 (*p* = 4.26 × 10^−15^), and that between MYL4 and type 17 T helper cells was 0.767121048 (*p* = 1.2 × 10^−12^). Meanwhile, the negative correlation between HBM and MYL4 and central memory CD4 T cells was also most prominent, the correlation between HBM and central memory CD4 T cells was −0.669661223 (*p* = 4.45 × 10^−12^), the correlation between MYL4 and central memory CD4 T cells was −0.6410939999621 (*p* = 6.64 × 10^−11^) (Figure 5(F)).

### 3.6. Construction of network relationship between biomarker, microRNA, lncRNA, and diseases

**Figure 6.**
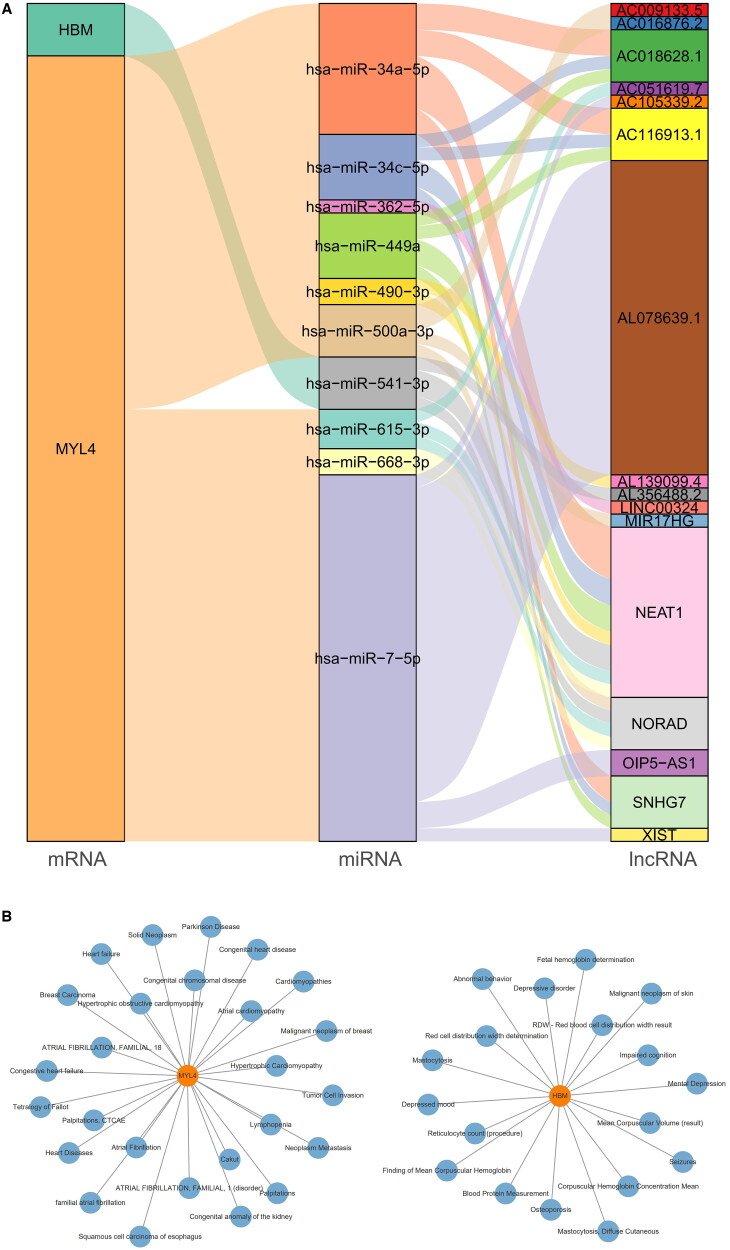
Regulation of molecular networks. **(A)** lncRNA-miRNA-biomarker network sankey diagram. Each rectangle represents a gene, with its connectivity visualized by the rectangle size. **(B)** Disease association analysis. Orange dots represent biomarkers, and blue dots represent potentially associated diseases. The left panel shows the MYL4 disease association network, and the right panel shows the HBM disease association network.

A lncRNA-miRNA-mRNA (biomarker) network was constructed and a total of 10 miRNAs (has-miR-34a-5p, has-miR-34c-5p, has-miR-362-5p, among other) and 16 lncRNAs (NEAT1, NORAD, ANHG7, among others) were found to potentially interact with the biomarkers HBM and MYL4 (Figure 6(A)). Notably, the biomarker HBM is specifically linked to only 1 miRNA and 3 lncRNAs, suggesting a selective and targeted regulatory relationship within this network.

The biomarker-disease association network revealed that the HBM network was associated with 19 diseases, such as malignant neoplasm of skin, mental depression, mastocytosis, and mastocytosis, diffuse cutaneous. MYL4 has been shown to be associated with 27 diseases in the MYL4 Network, including atrial fibrillation, malignant neoplasm of breast (breast cancer), neoplasm metastasis, squamous cell carcinoma of esophagus, solid neoplasm, breast carcinoma, cardiomyopathies, and tumor cell Invasion, among others. These associations highlight the potential involvement of MYL4 in a wide spectrum of pathological conditions (Figure 6(B)).

### 3.7. Expression validation of HBM and MYL4

**Figure 7.**
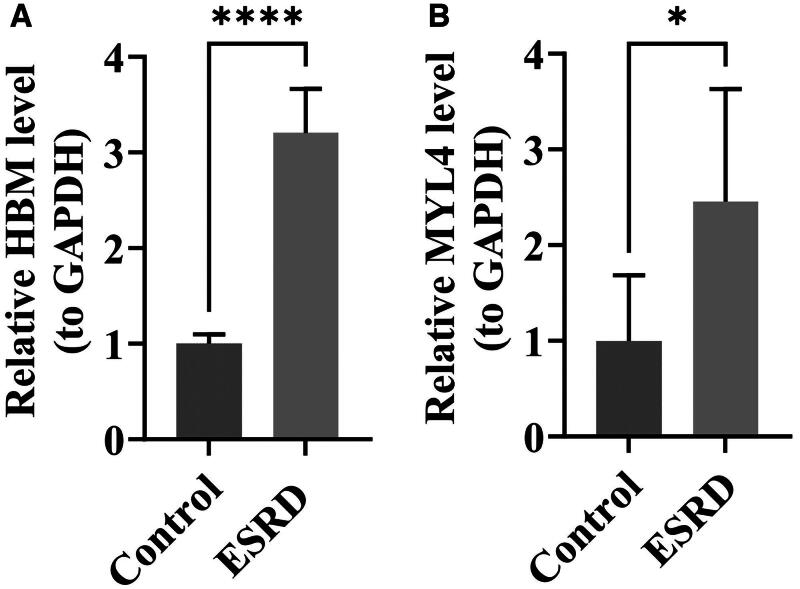
Results of expression analysis. **(A)** Expression analysis of HBM. **** indicates *p* < 0.0001. **(B)** Expression analysis of MYL4. * indicates *p* < 0.05.

RT-qPCR validation of clinical samples showed that the expression of the HBM and MYL4 genes in ESRD was significantly higher than that of controls (Figure 7(A,B)) (*p* < 0.05). The expression patterns of these two biomarkers in ESRD aligned with the findings from bioinformatics analysis.

## 4. Discussion

The global prevalence of ESRD is progressively increasing. Due to the numerous comorbidities, diverse pathogenic factors, complex mechanisms of onset, and difficulty in early screening, there are currently no effective, targeted treatments for ESRD. Additionally, patients require long-term maintenance treatment, which can lead to premature loss of social functioning, imposing a heavy burden on affected families and society. Parthanatos, a type of programmed cell death, has been found in previous studies to be alleviated by the lack of its key initiator, PARP1, which can reduce renal fibrosis and mitigate renal dysfunction [29]. However, the molecular mechanism of PRGs in ESRD remains unclear. Therefore, this study identified biomarkers related to parthanatos in ESRD, specifically MYL4 and HBM, through bioinformatics screening and constructed a diagnostic model.

HBM is a protein associated with cell adhesion and signal transduction, whose molecular structure includes various proteins, lipids, and carbohydrates. These components work together to influence cell adhesion, proliferation, and migration [30]. Current research has found that HBM can affect the function of renal tubules through various signaling pathways, mainly including the PI3K/Akt, MAPK, and NF-κB pathways. These signaling pathways play a key role in regulating renal inflammation and fibrosis. For example, the activation of the PI3K/Akt pathway can promote cell survival and proliferation [31], the MAPK pathway is closely related to cellular stress responses and proliferation [32], and the activation of the NF-κB pathway is closely related to the regulation of inflammatory responses [33]. In ESRD, the upregulation of HBM may promote the proliferation and survival of renal tubular cells through the activation of these signaling pathways, thereby exacerbating the progression of the pathological process [34]. Additionally, HBM may also influence the renal microenvironment by regulating the release of cytokines, further aggravating kidney damage. Moreover, the upregulation of HBM is closely related to the occurrence of renal inflammation. Studies have shown that certain components in HBM can stimulate renal tubular cells to release inflammatory factors, such as tumor necrosis factor-alpha (TNF-α) and interleukin-6 (IL-6), which can further trigger and sustain renal inflammatory responses [35]. Therefore, the mechanism of action of HBM in ESRD involves not only intercellular interactions but also the regulation of inflammatory responses, which may provide new ideas and strategies for the treatment of ESRD.

MYL4 is a small subunit of actin, primarily involved in maintaining cell morphology and movement. Research has shown that the role of MYL4 in cardiac and muscle tissues has been widely explored; however, its mechanism of action in the kidneys remains to be studied in depth. The expression level of MYL4 is associated with various cardiovascular diseases, particularly in the processes of atrial fibrosis and cardiac remodeling, where its function is particularly important [36]. Studies have found that increased expression of MYL4 may be related to the progression of renal fibrosis, suggesting that it may play an important role in the pathophysiology of ESRD [37]. The interaction of MYL4 with the connexin protein Cx43 is believed to play an important role in atrial fibrosis, and this mechanism may also apply to the study of renal fibrosis [37]. By regulating the expression and function of MYL4, new targets and strategies for the treatment of ESRD may be provided. Therefore, in-depth research on the mechanism of action of MYL4 in renal cells will help us better understand the pathophysiological processes of ESRD and provide a theoretical basis for developing new treatment methods [38,39].

GSEA enrichment analysis indicates that both HBM and MYL4 are co-enriched in pathways such as Natural Killer (NK) Cell-Mediated Cytotoxicity and T Cell Receptor Signaling Pathway. NK cell cytotoxicity refers to the process by which NK cells selectively release lytic granules or induce apoptosis through the expression of Fas ligand, thereby killing infected or transformed cells [40]. Research has shown that MYL4 may also regulate cell survival and death by affecting the intracellular autophagy process, a mechanism that plays an important role in the occurrence and development of kidney diseases [41]. Dysregulation of MYL4 may lead to impaired autophagic flux, thereby promoting cell fibrosis and apoptosis [42]^.^ These studies suggest that the Natural Killer Cell-Mediated Cytotoxicity pathway may influence the progression of ESRD by regulating apoptosis. The T Cell Receptor (TCR) Signaling Pathway is a complex network that controls the activation and function of T cells. It recognizes and binds antigen peptides presented on the surface of antigen-presenting cells through the T cell receptor on the T cell surface [43,44]. Existing studies indicate that toxins in ESRD may interfere with the normal function of protein tyrosine kinases, thereby affecting normal TCR signaling transduction, resulting in abnormal T cell activation, proliferation, and differentiation. Abnormal TCR signaling may alter the immunoregulatory functions of T cells, affecting the release of inflammatory factors, which in turn influences the local renal microenvironment and promotes further kidney tissue damage, creating a vicious cycle [45]. Certain components in HBM can promote the proliferation of regulatory T cells, thereby inhibiting the excessive activation of inflammatory responses. Based on these findings, it can be inferred that the T Cell Receptor Signaling Pathway may play a role in ESRD [46]. Therefore, HBM and MYL4 may influence the development of ESRD by participating in the Natural Killer Cell-Mediated Cytotoxicity and T Cell Receptor Signaling Pathway, making them potential biomarkers for ESRD.

The relationship between kidney disease and the infiltration of immune cells is complex. In acute kidney injury (AKI) and chronic kidney disease (CKD), the infiltration of immune cells is considered an important factor leading to kidney damage and loss of function. Studies have shown that there is often a significant infiltration of immune cells in the kidneys of AKI patients, particularly macrophages and T cells, and this infiltration is related to the severity of kidney damage [47]. Additionally, the kidneys of CKD patients also exhibit significant immune cell infiltration, which is closely associated with chronic inflammation and fibrosis in the kidneys. Therefore, research on immune cell infiltration in kidney diseases not only helps to understand the mechanisms of disease occurrence but may also provide a basis for new therapeutic strategies [48]^.^ Correlation analysis between biomarkers and immune cells shows that HBM and MYL4 have the strongest positive correlation with 17 T helper cells and the strongest negative correlation with central memory CD4 T cells. Interleukin 17 (IL-17) is a pro-inflammatory cytokine known to provide defense against various microorganisms and play a pathogenic role in many autoimmune diseases [49]. The inflammatory response is critically important in various kidney diseases, with renal perivascular cells (pericytes/fibroblasts) producing pro-inflammatory molecules, promoting immune cell infiltration, and enhancing the inflammatory response [50]. Thus, 17 T helper cells may contribute to ESRD progression through their pro-inflammatory response. Additionally, studies have shown that CD4(+) T cells are involved in initiating T cell differentiation, potentially programming epigenetic qualitative differences into ensuing effector and memory populations. Defining the memory qualities that best protect against re-infection, as well as how commitment to the memory lineage is determined following T-cell activation, remains an important goal. In patients undergoing peritoneal dialysis and hemodialysis, CD4+ levels differ, possibly due to differences in inflammatory markers associated with different dialysis methods [51]. This suggests that central memory CD4 T cells and ESRD are closely related. This further suggests that HBM and MYL4 may play a role in ESRD through these two immune cells and may be potential biomarkers for ESRD. Current research indicates that HBM may influence the inflammatory response in the kidneys by regulating the activity of immune cells. Certain components in HBM can promote the proliferation of regulatory T cells, thereby inhibiting the excessive activation of the inflammatory response, a mechanism that is of significant importance in the pathophysiology of ESRD [46]. The expression and activity of MYL4 may be regulated by the signaling pathways activated by HBM, thereby enhancing the anti-apoptotic ability of renal tubular cells. Research indicates that HBM enhances the resistance of cells to apoptotic stimuli by promoting the expression of MYL4, which may play an important role in the development of ESRD [52]. In addition, drug therapies that inhibit immune cell infiltration and gene therapy as emerging treatment strategies can suppress the abnormal infiltration of immune cells by directly regulating their functions and behaviors. This study aims to provide new insights for the treatment of end-stage renal disease through a preliminary exploration of the correlation between the HBM and MYL4 gene loci and ESRD.

In the lncRNA-miRNA-biomarker network, HBM and MYL4 were predicted to target miRNA (hsa-miR-541-3p) and lncRNA (NEAT1). Currently, there is no research linking hsa-miR-541-3p to kidney diseases, and its potential role in kidney pathology requires further investigation. Nuclear-enriched abundant transcript 1 (NEAT1) is a long non-coding RNA (lncRNA) that is widely expressed in mammalian cells. In recent years, increasing studies have shown that lncRNA NEAT1 is significantly upregulated in various organ fibrosis processes, including liver fibrosis, renal fibrosis, cardiac fibrosis, and pulmonary fibrosis. Existing research demonstrates that knocking down NEAT1 significantly reduces fibrosis both *in vitro* and *in vivo*. One study revealed that NEAT1 is induced in STZ-induced diabetic kidneys, and its expression is notably suppressed following shRNA knockdown. Compared to non-diabetic controls, diabetic mice exhibited increased UACR, with significant tubular dilation and atrophy. However, NEAT1 knockdown alleviated proteinuria and kidney damage in diabetic mice, indicating that elevated Neat1 expression contributes to the development of diabetic kidney disease (DKD) [53]. The above studies suggest that NEAT1 influences the progression of ESRD to some extent.

However, due to the small sample size in public databases, there may be selection bias, and relying solely on PCR results cannot fully prove the causal relationship between HBM and MYL4 gene expression and the pathogenesis of ESRD. Nevertheless, the results of this study lay the foundation for future experimental research with larger sample sizes. In the future, we can further increase the sample size by collecting data from multiple centers, expanding samples from different ethnic groups, and including clinical samples of end-stage renal disease, to verify the accuracy and specificity of HBM and MYL4 as biomarkers of renal function injury, aiming to provide more evidence for the early identification of high-risk patients and timely measures to slow disease progression and early diagnosis. Additionally, we hope to further explore whether HBM and MYL4 genes directly participate in the pathological process of ESRD through *in vitro* cell experiments or animal model experiments, focusing on the interactions of these two factors in the renal microenvironment and their effects on cellular signal transduction. By studying processes such as inflammatory responses, cellular fibrosis, or apoptosis, we aim to further investigate their specific molecular mechanisms and signaling pathways, with the goal of providing new biomarkers and targeted therapeutic strategies for the early diagnosis and treatment of ESRD. Furthermore, studies have conducted bibliometric analyses of the application of multi-omics in drug discovery, revealing major trends and research hotspots in this field. Research indicates that multi-omics has significant potential in drug discovery, particularly in predicting drug sensitivity [54]. With technological advancements, the application potential of multi-omics will continue to grow, and in the future, intelligent screening of factors related to end-stage renal disease through multi-omics analysis is expected to accelerate the drug discovery process, which will have a profound impact on disease treatment and improving patient survival rates.

This study conducted a correlation analysis between parthanatos-related genes and ESRD, demonstrating the association between parthanatos-related genes and chronic kidney diseases ESRD. In the pathophysiology of ESRD, HBM and MYL4 may have significant specific potential. This specificity mainly arises from the unique microenvironment of the kidneys and the influence of these factors on cellular signal transduction. The expression and function of HBM and MYL4 in the kidneys show significant differences compared to other tissues, providing new perspectives for understanding the pathogenesis of ESRD. Overall, the specific mechanisms of HBM and MYL4 in ESRD offer new research directions, with HBM and MYL4 showing potential as possible biomarkers in ESRD, providing new avenues for further research on the role of parthanatos in the mechanisms of ESRD.

## Supplementary Material

Supplementary Figure 1 Analysis flowchart of this study.tif

_.zip

## Data Availability

The data that support the findings of this study are openly available in [Gene Expression Omnibus] at [https://www.ncbi.nlm.nih.gov/geo/], reference number [GSE37171 and GSE142153].
