# Supplementary figures and images for: Identification and validation of parthanatos-related genes in end-stage renal disease
Source: Ren Fail. 2025 Jul 6;47(1):2519834. doi: 10.1080/0886022X.2025.2519834 (PMC12231245; doi:10.1080/0886022X.2025.2519834)

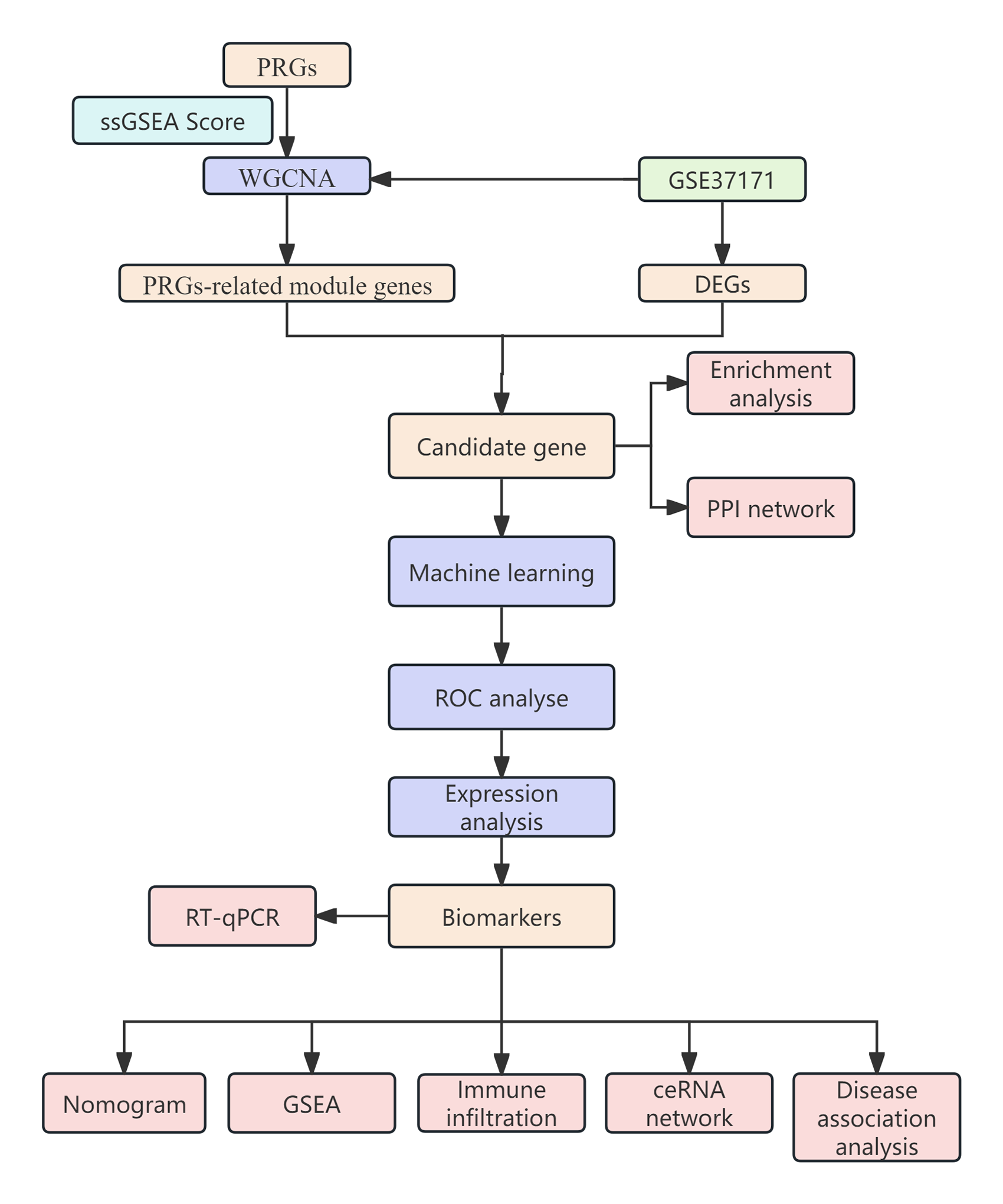

Supplement: Supplementary Figure 1 Analysis flowchart of this study.tif [file IRNF_A_2519834_SM0593.tif]

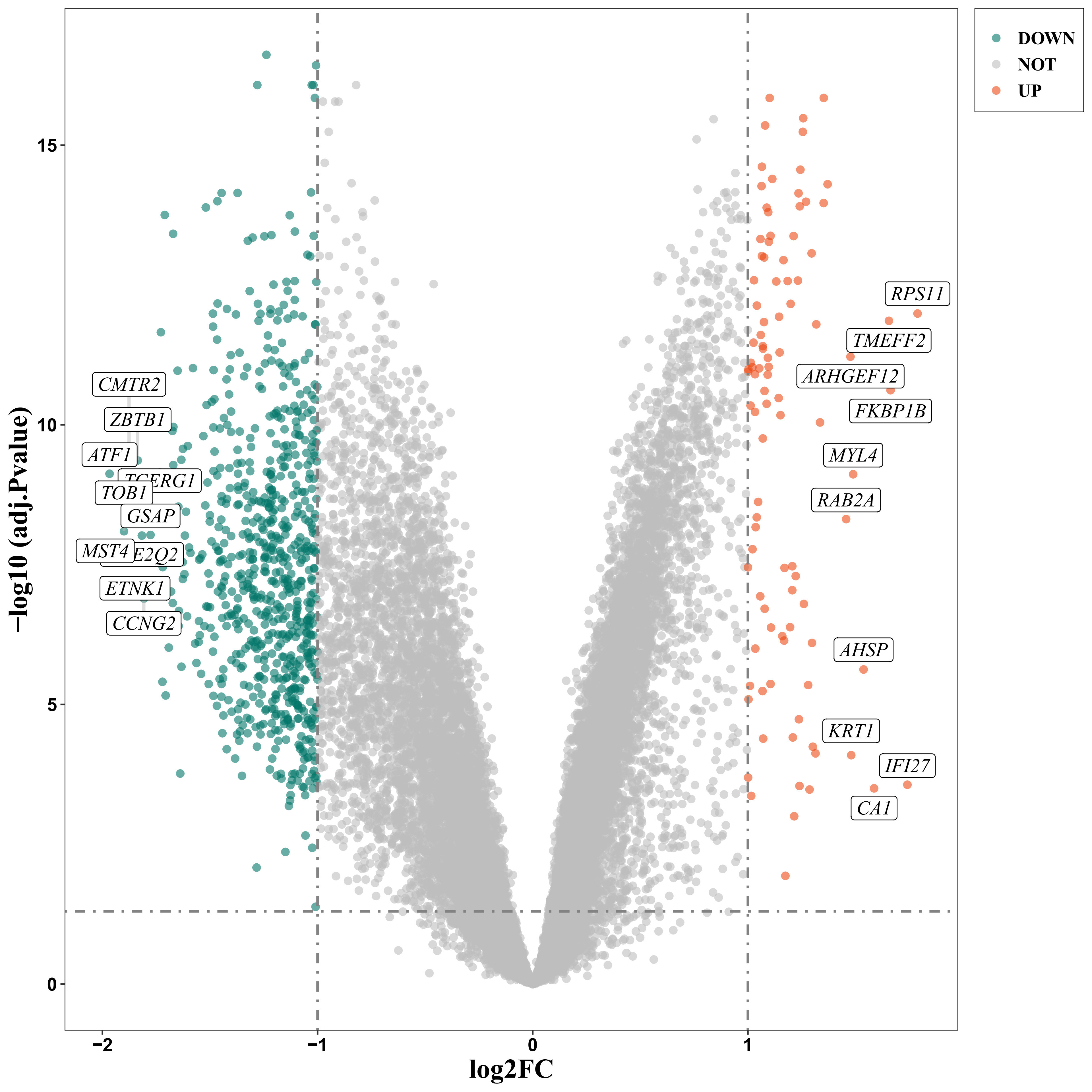

Supplement: _.zip [file IRNF_A_2519834_SM0592.zip › 图片终稿/Figure 1A.jpg]

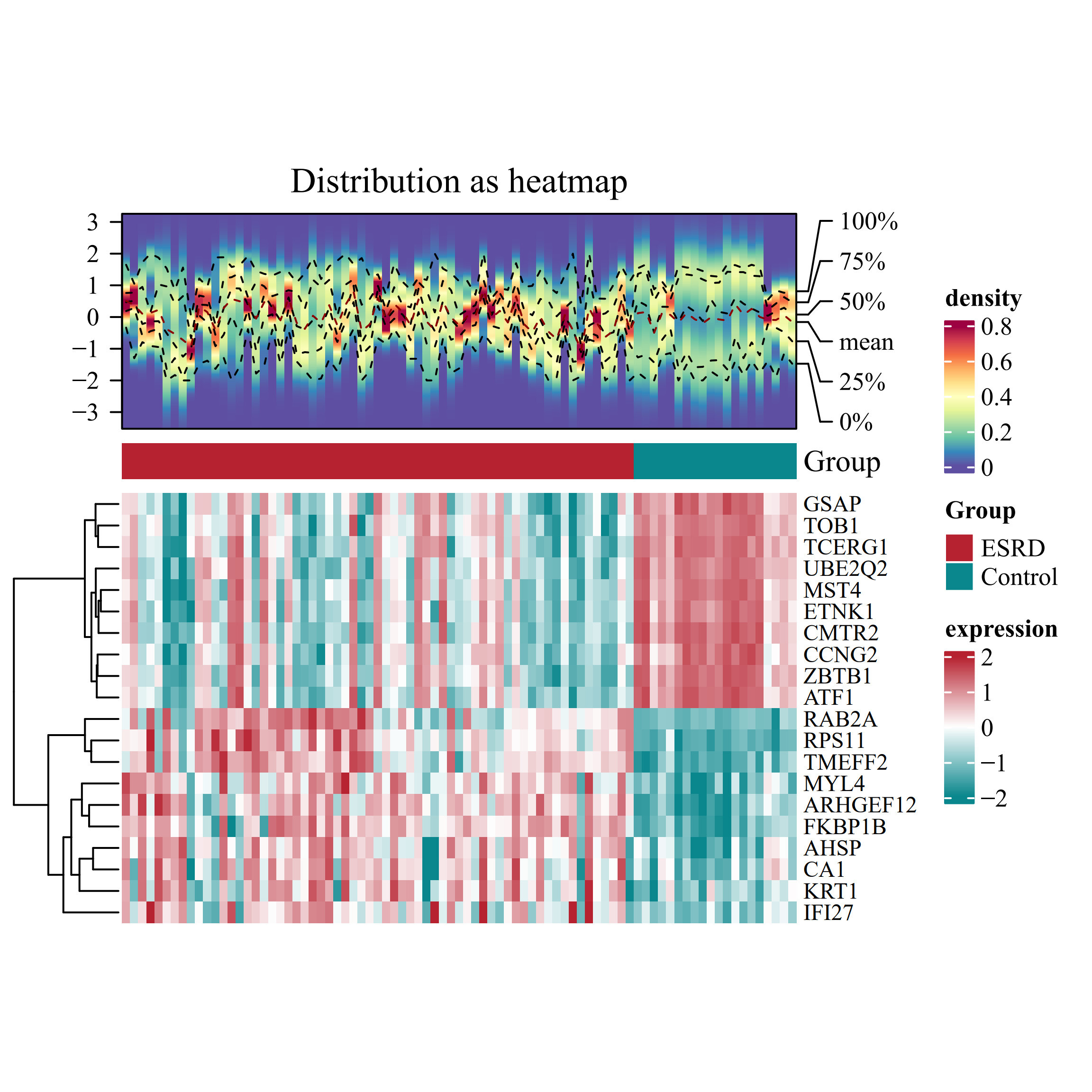

Supplement: _.zip [file IRNF_A_2519834_SM0592.zip › 图片终稿/Figure 1B.jpg]

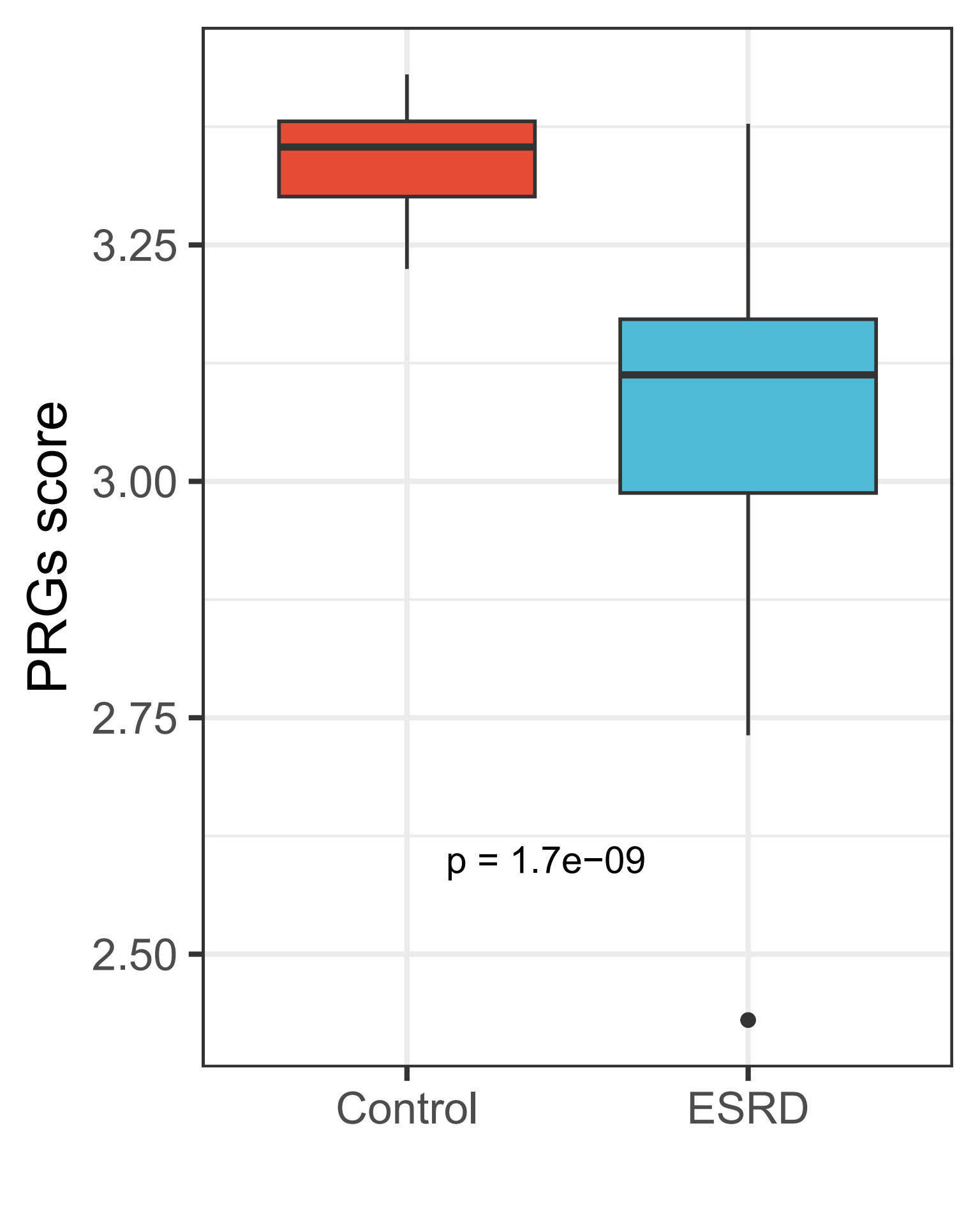

Supplement: _.zip [file IRNF_A_2519834_SM0592.zip › 图片终稿/Figure 1C.jpg]

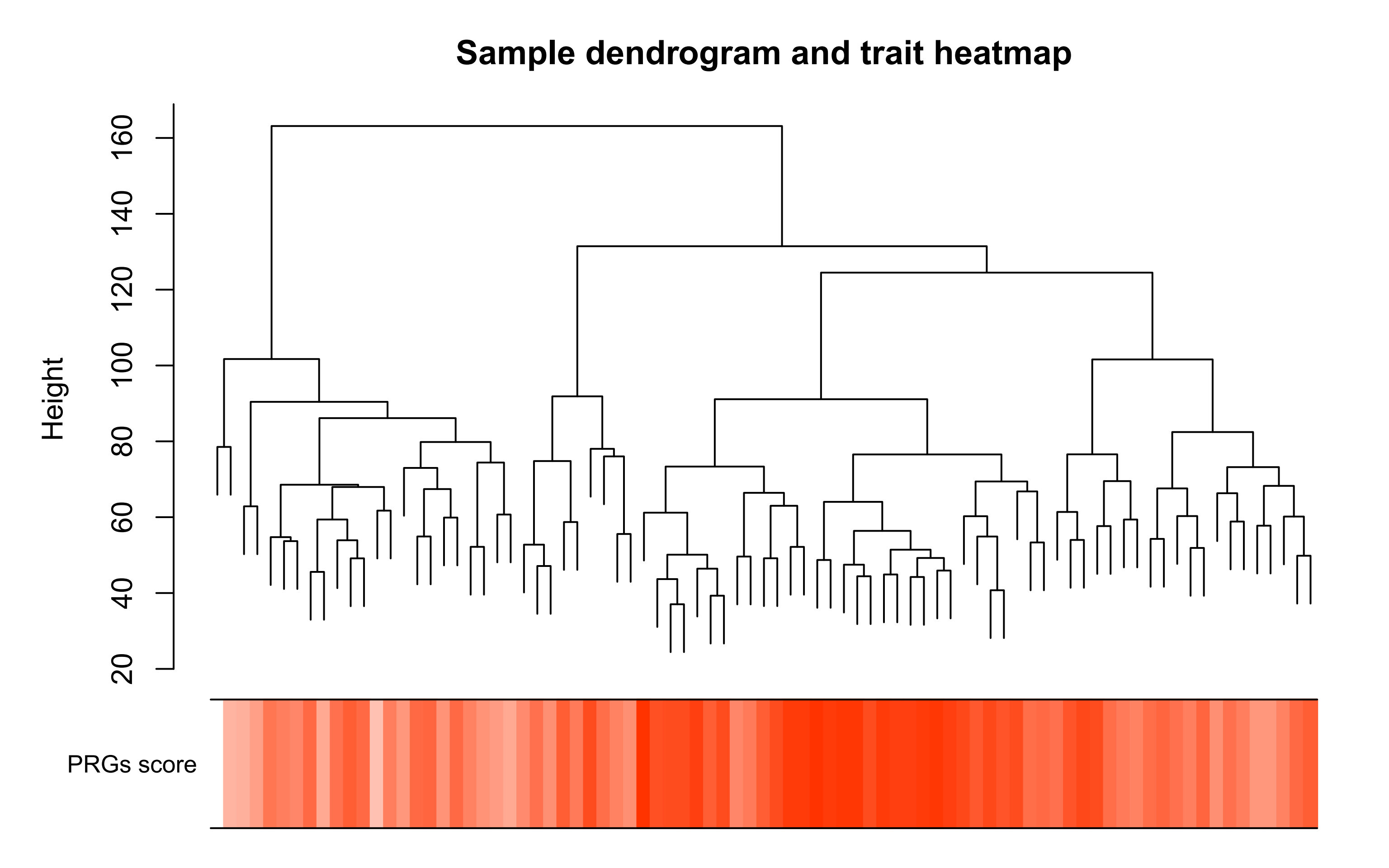

Supplement: _.zip [file IRNF_A_2519834_SM0592.zip › 图片终稿/Figure 1D.jpg]

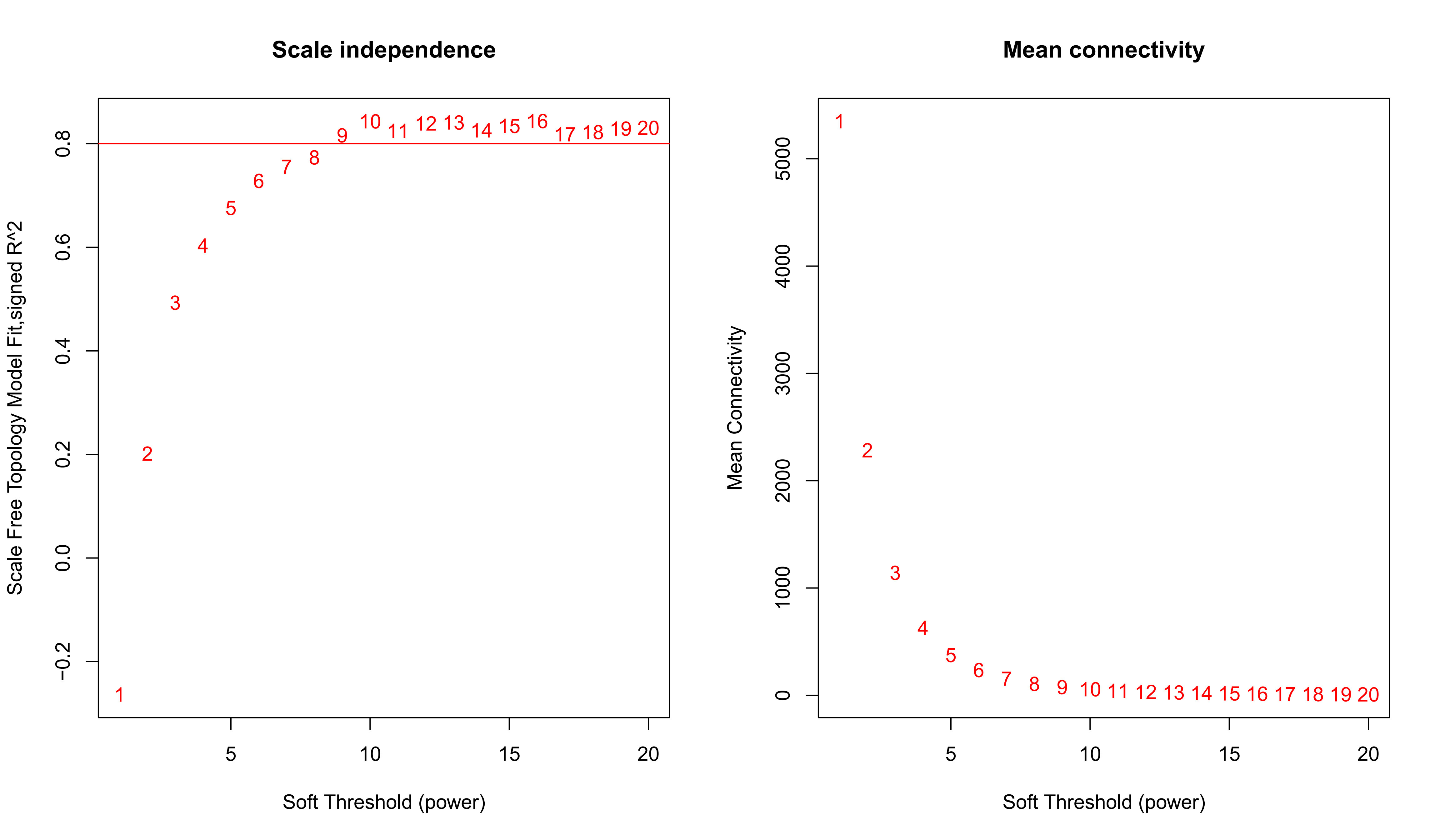

Supplement: _.zip [file IRNF_A_2519834_SM0592.zip › 图片终稿/Figure 1E.jpg]

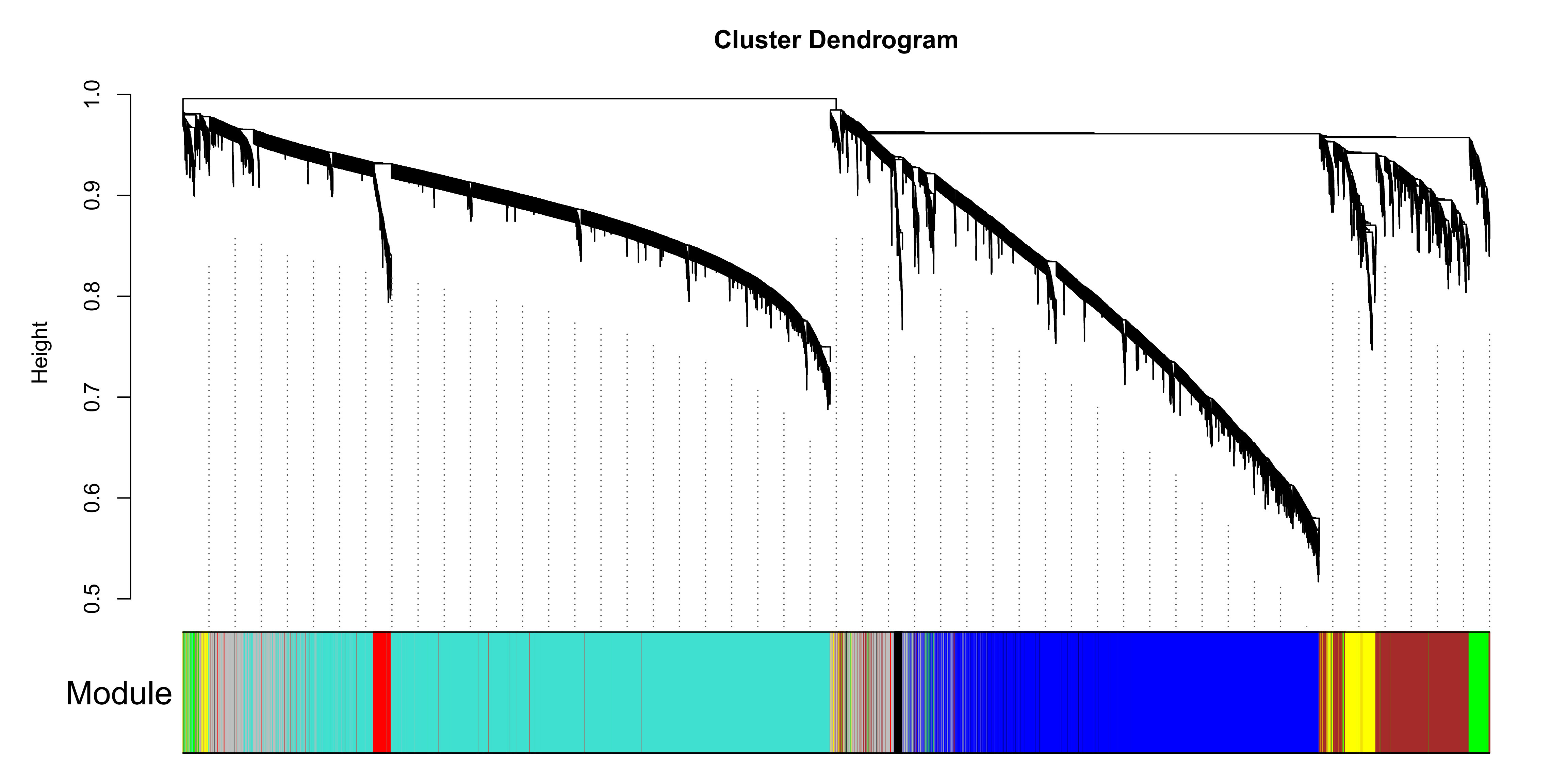

Supplement: _.zip [file IRNF_A_2519834_SM0592.zip › 图片终稿/Figure 1F.jpg]

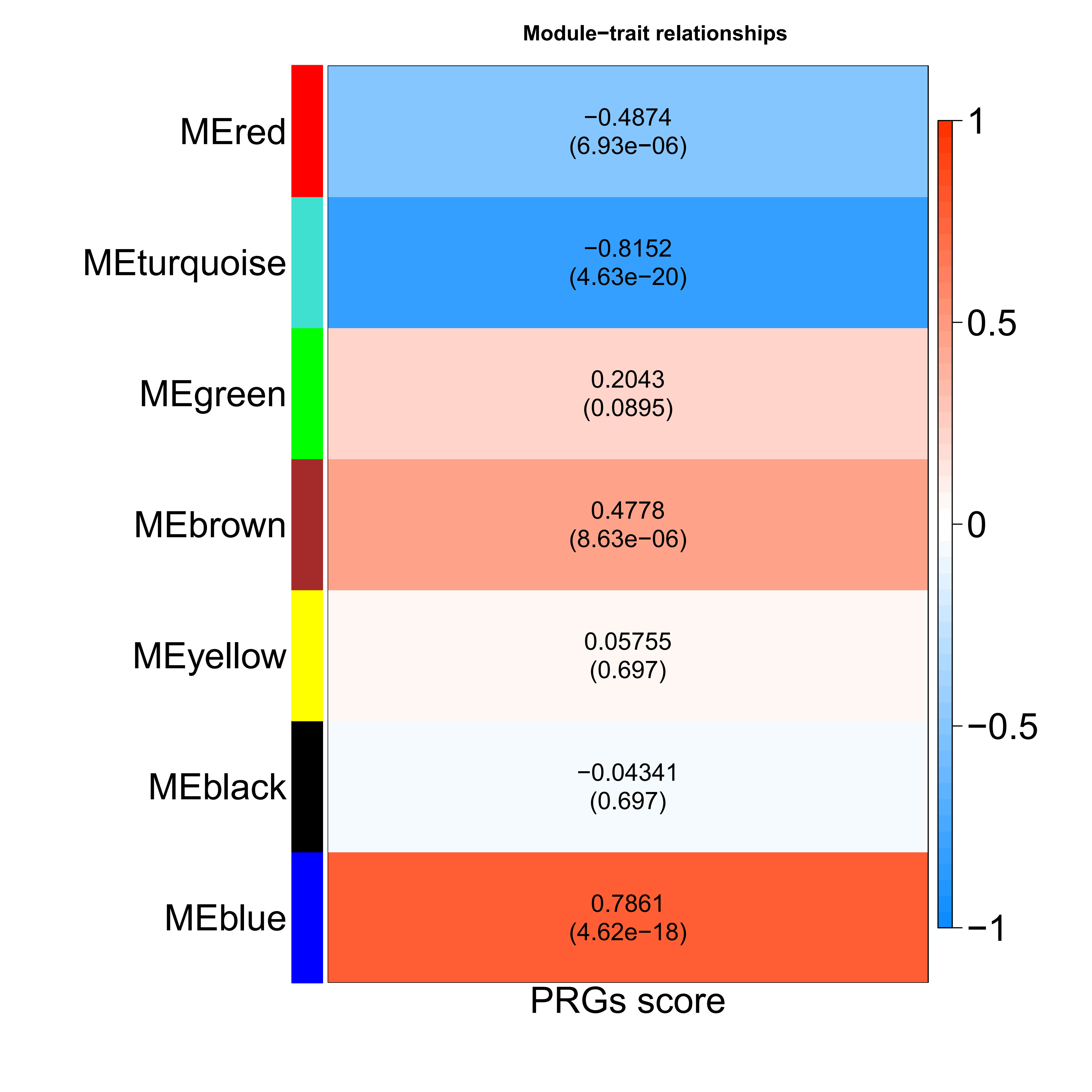

Supplement: _.zip [file IRNF_A_2519834_SM0592.zip › 图片终稿/Figure 1G.jpg]

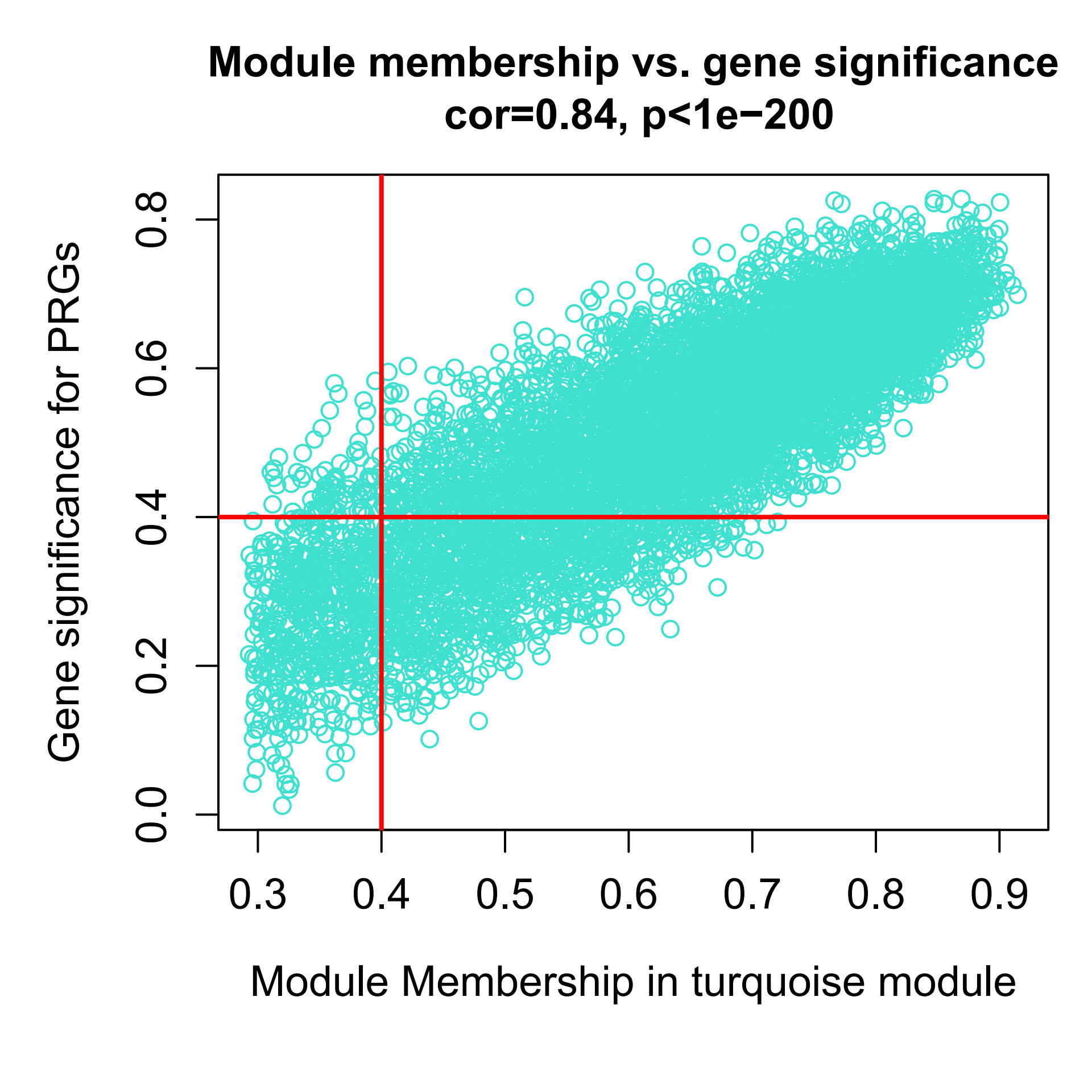

Supplement: _.zip [file IRNF_A_2519834_SM0592.zip › 图片终稿/Figure 1H.jpg]

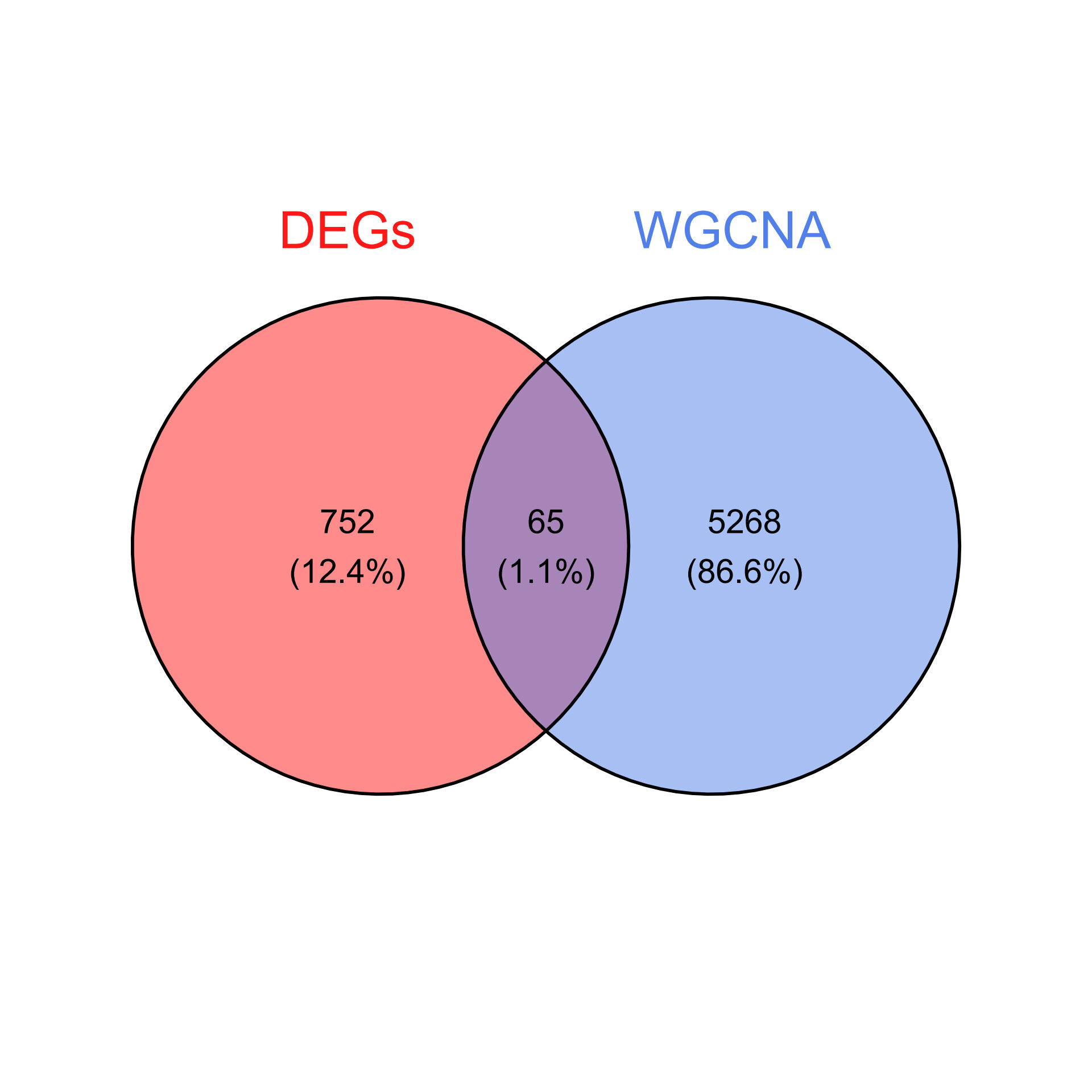

Supplement: _.zip [file IRNF_A_2519834_SM0592.zip › 图片终稿/Figure 1I.jpg]

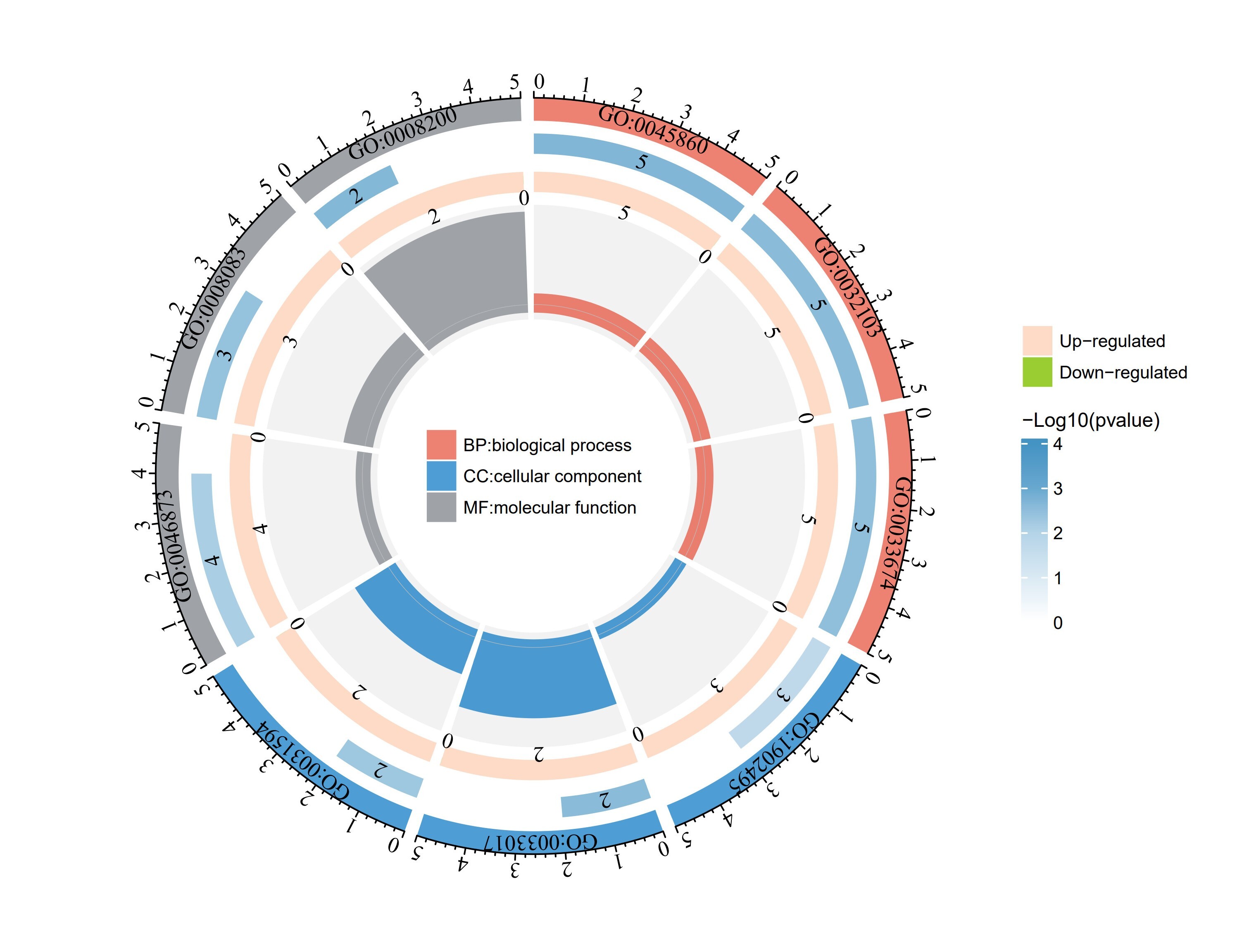

Supplement: _.zip [file IRNF_A_2519834_SM0592.zip › 图片终稿/Figure 2A.jpg]

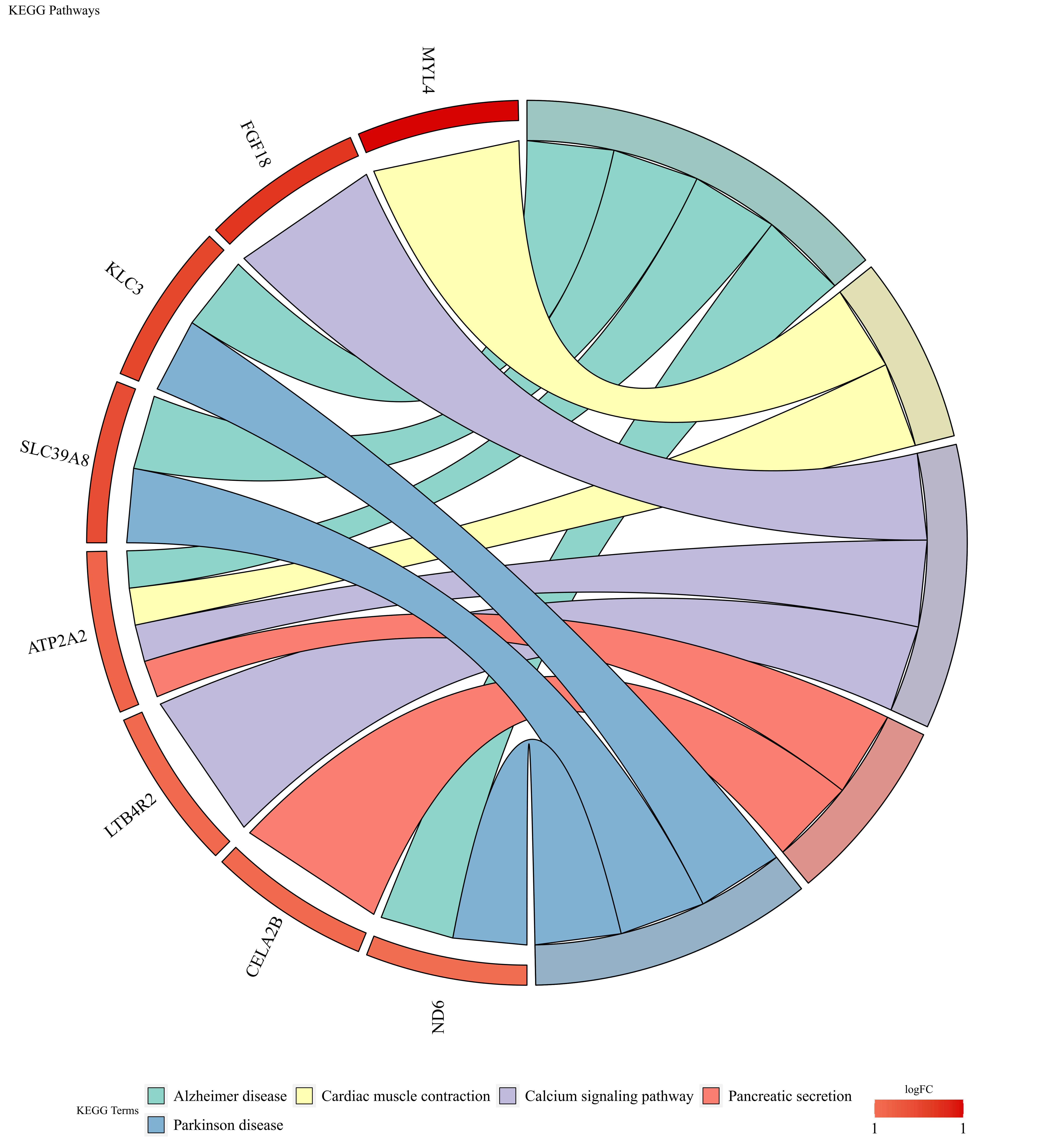

Supplement: _.zip [file IRNF_A_2519834_SM0592.zip › 图片终稿/Figure 2B.jpg]

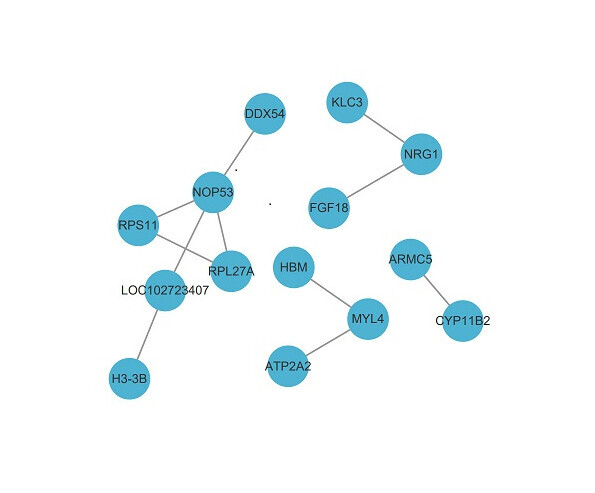

Supplement: _.zip [file IRNF_A_2519834_SM0592.zip › 图片终稿/Figure 2C.jpg]

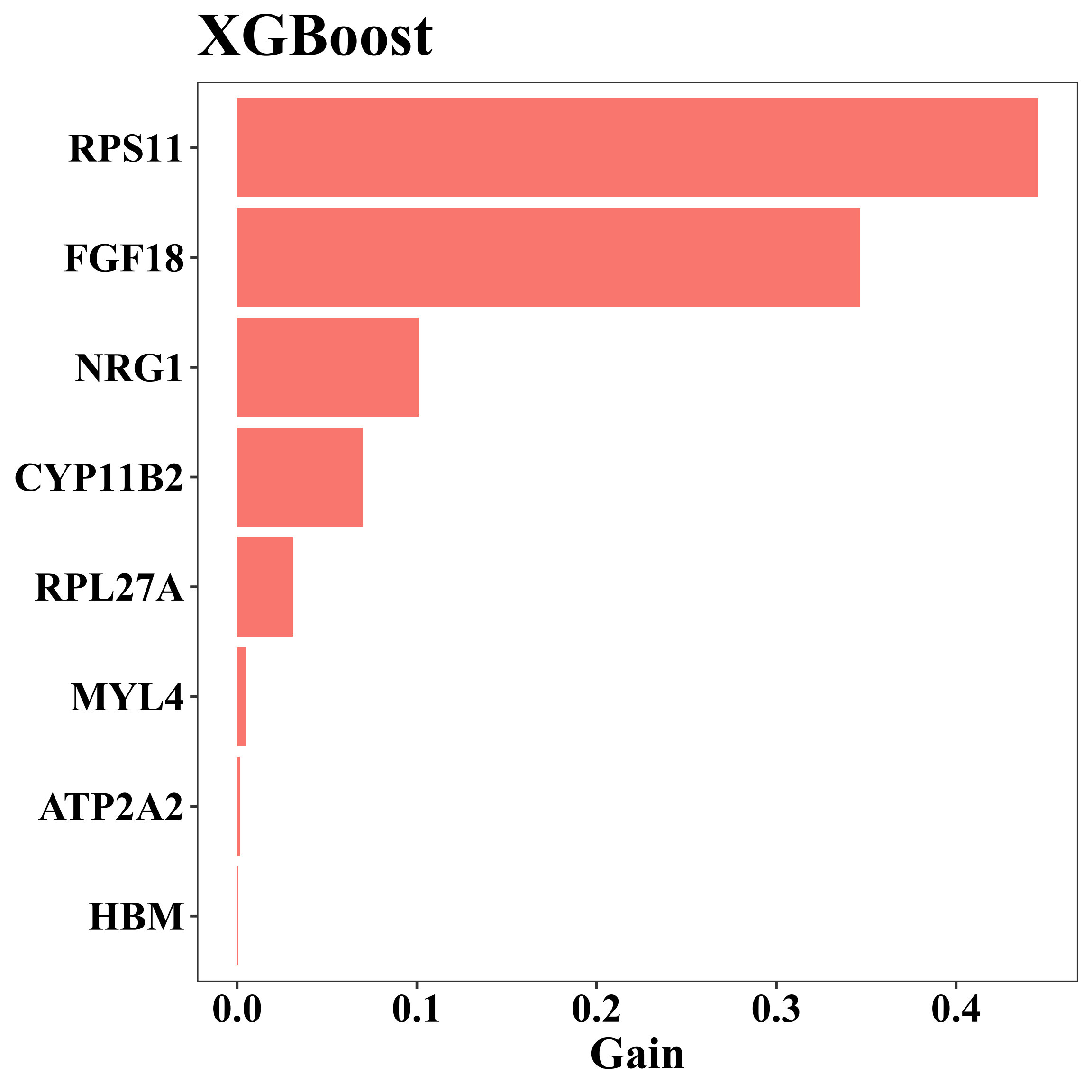

Supplement: _.zip [file IRNF_A_2519834_SM0592.zip › 图片终稿/Figure 3A.jpg]

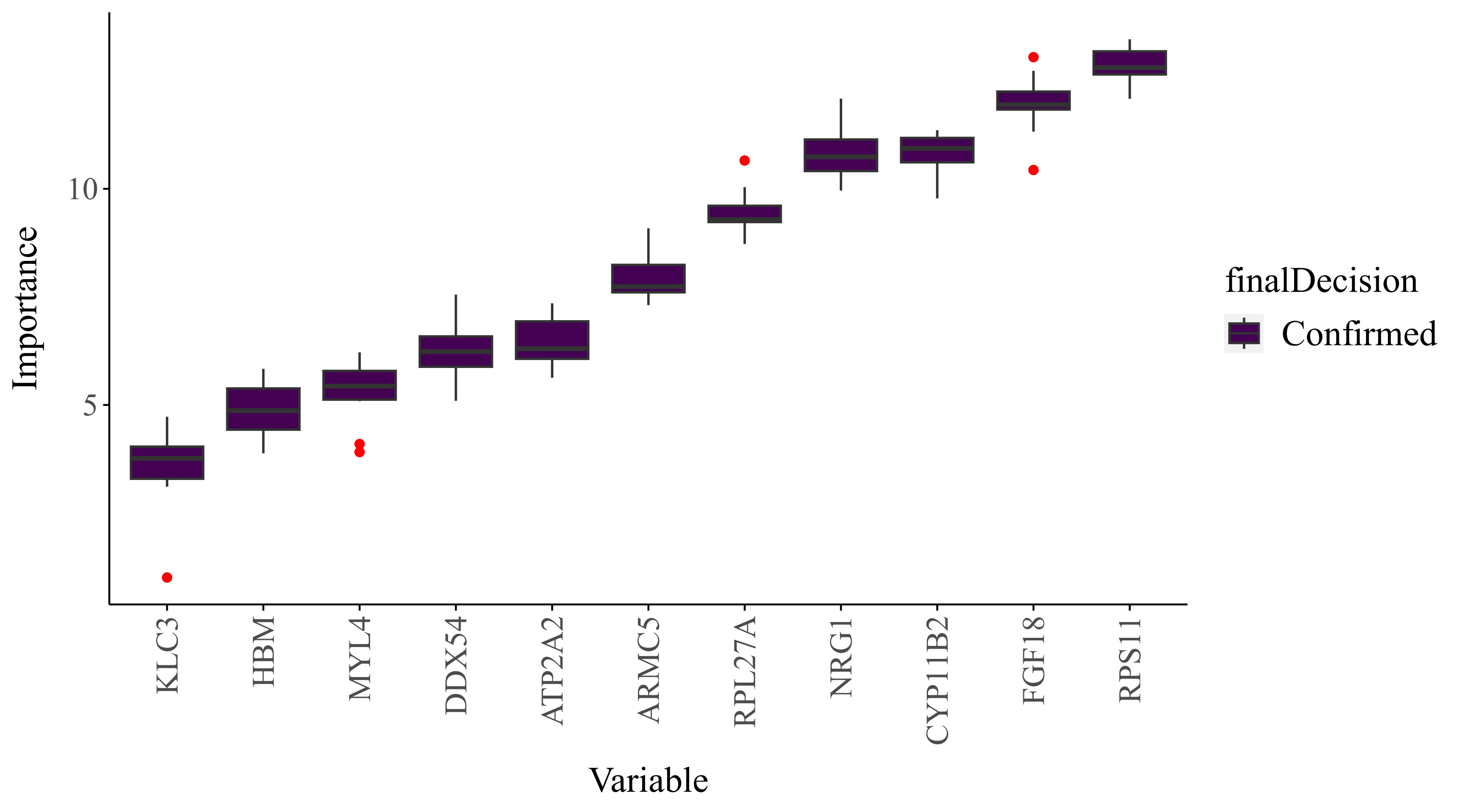

Supplement: _.zip [file IRNF_A_2519834_SM0592.zip › 图片终稿/Figure 3B.jpg]

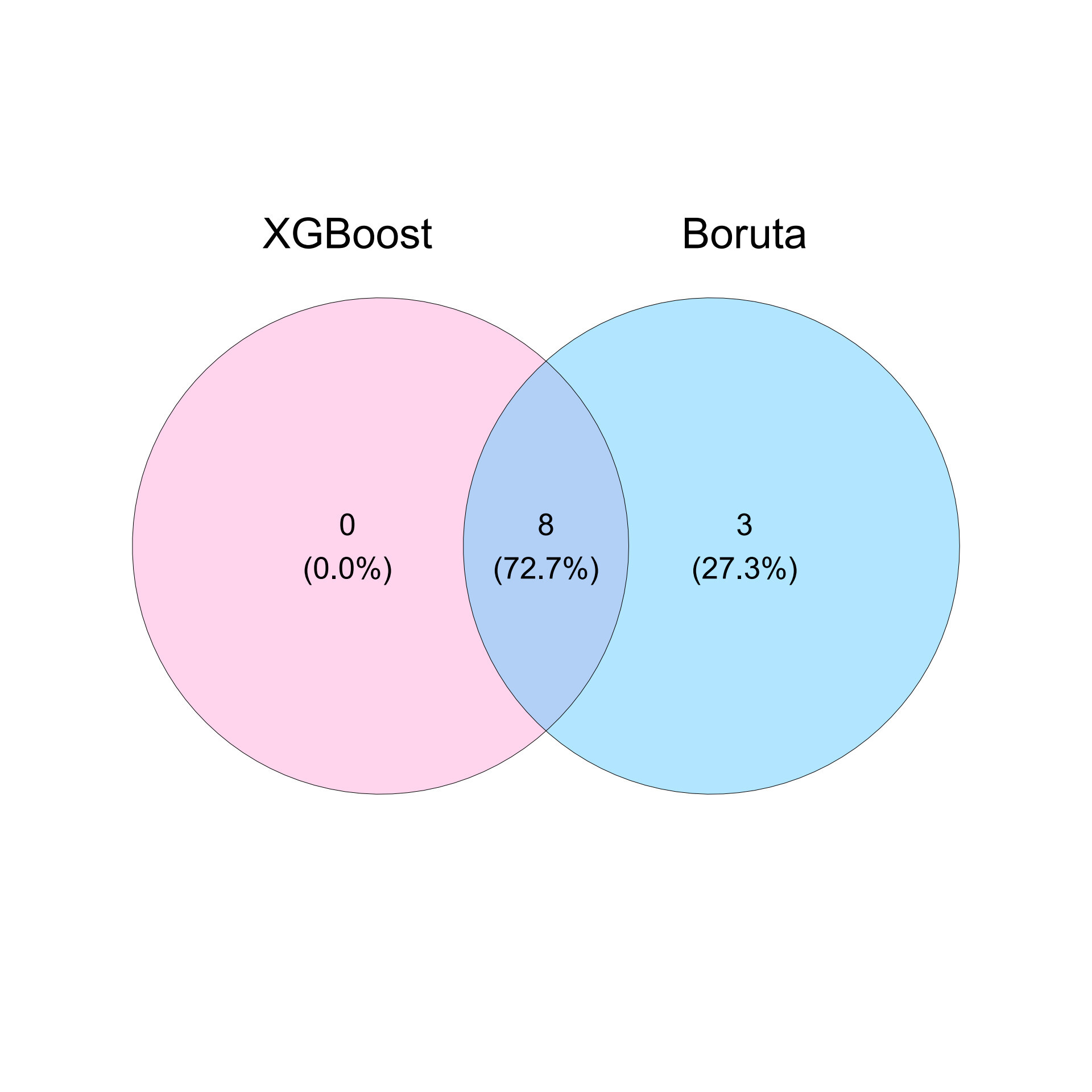

Supplement: _.zip [file IRNF_A_2519834_SM0592.zip › 图片终稿/Figure 3C.jpg]

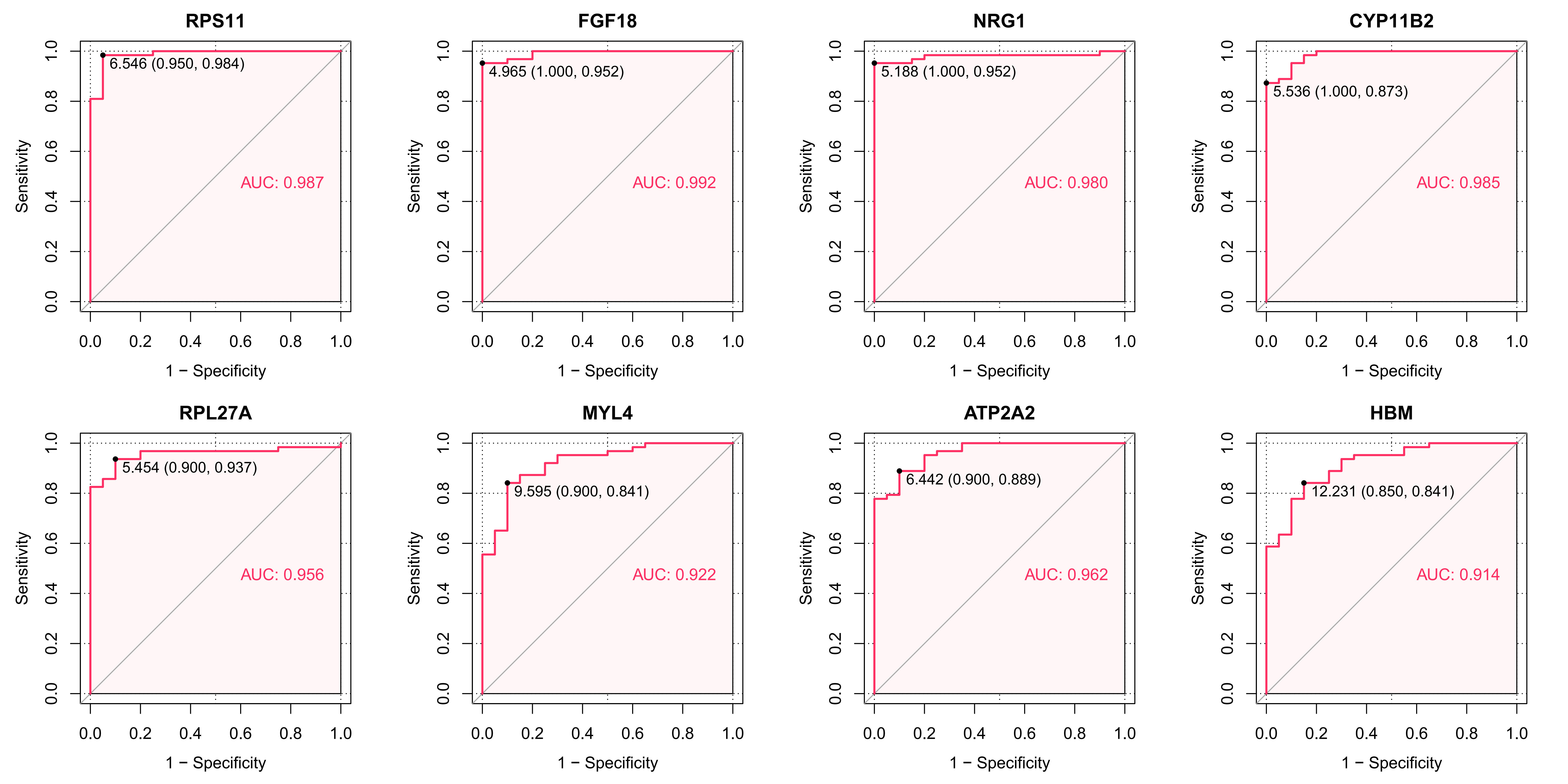

Supplement: _.zip [file IRNF_A_2519834_SM0592.zip › 图片终稿/Figure 4A.jpg]

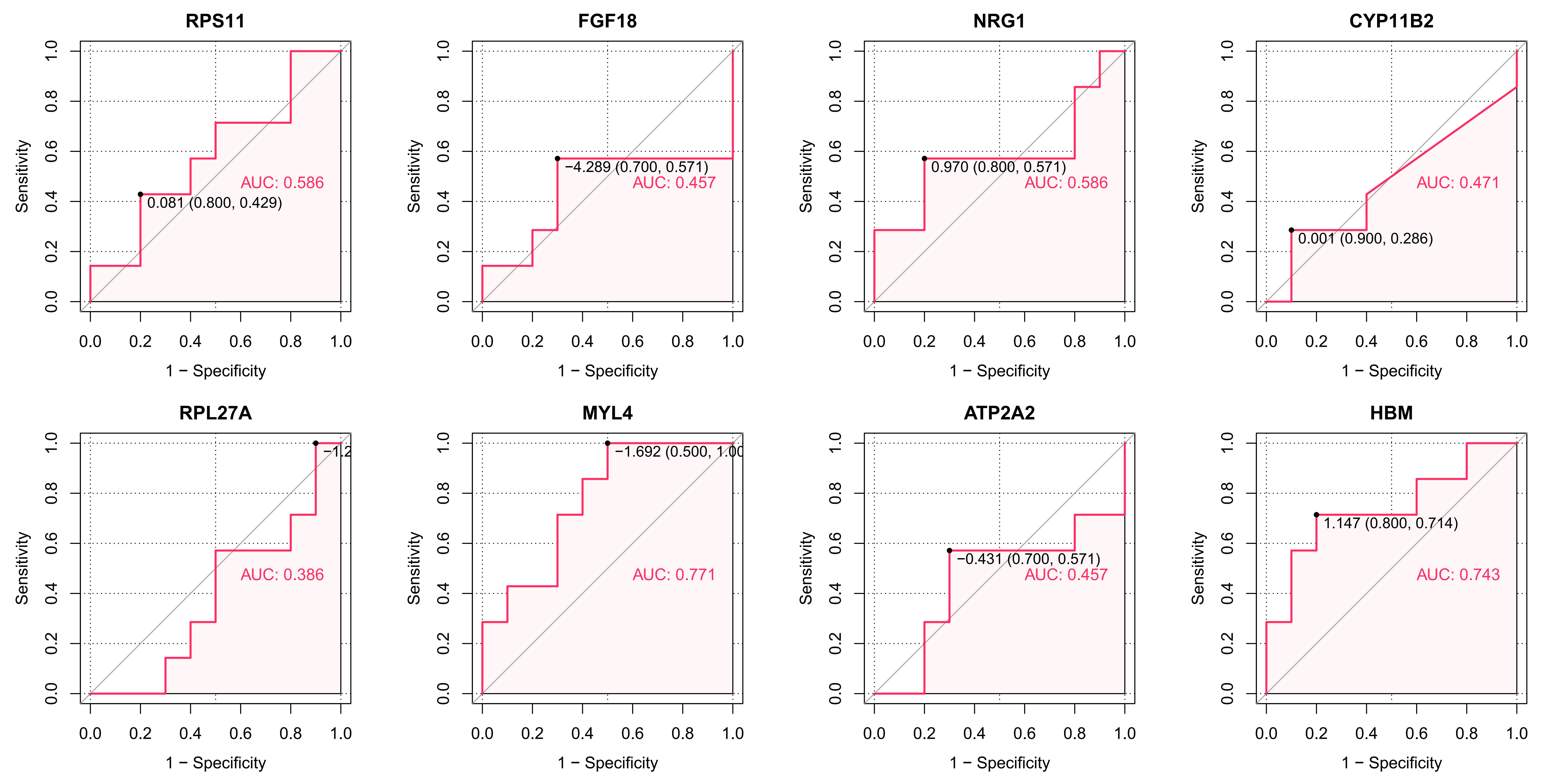

Supplement: _.zip [file IRNF_A_2519834_SM0592.zip › 图片终稿/Figure 4B.jpg]

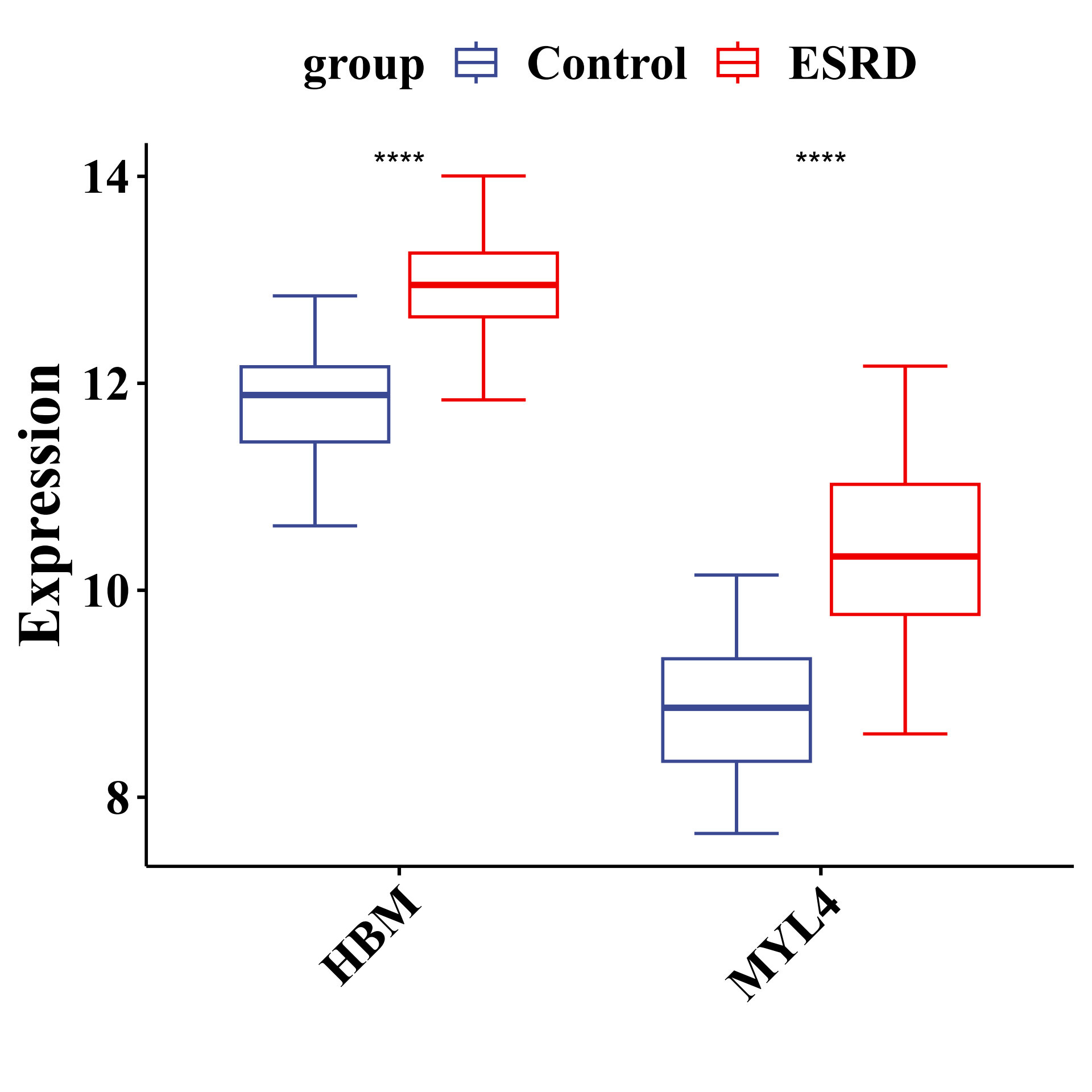

Supplement: _.zip [file IRNF_A_2519834_SM0592.zip › 图片终稿/Figure 4C.jpg]

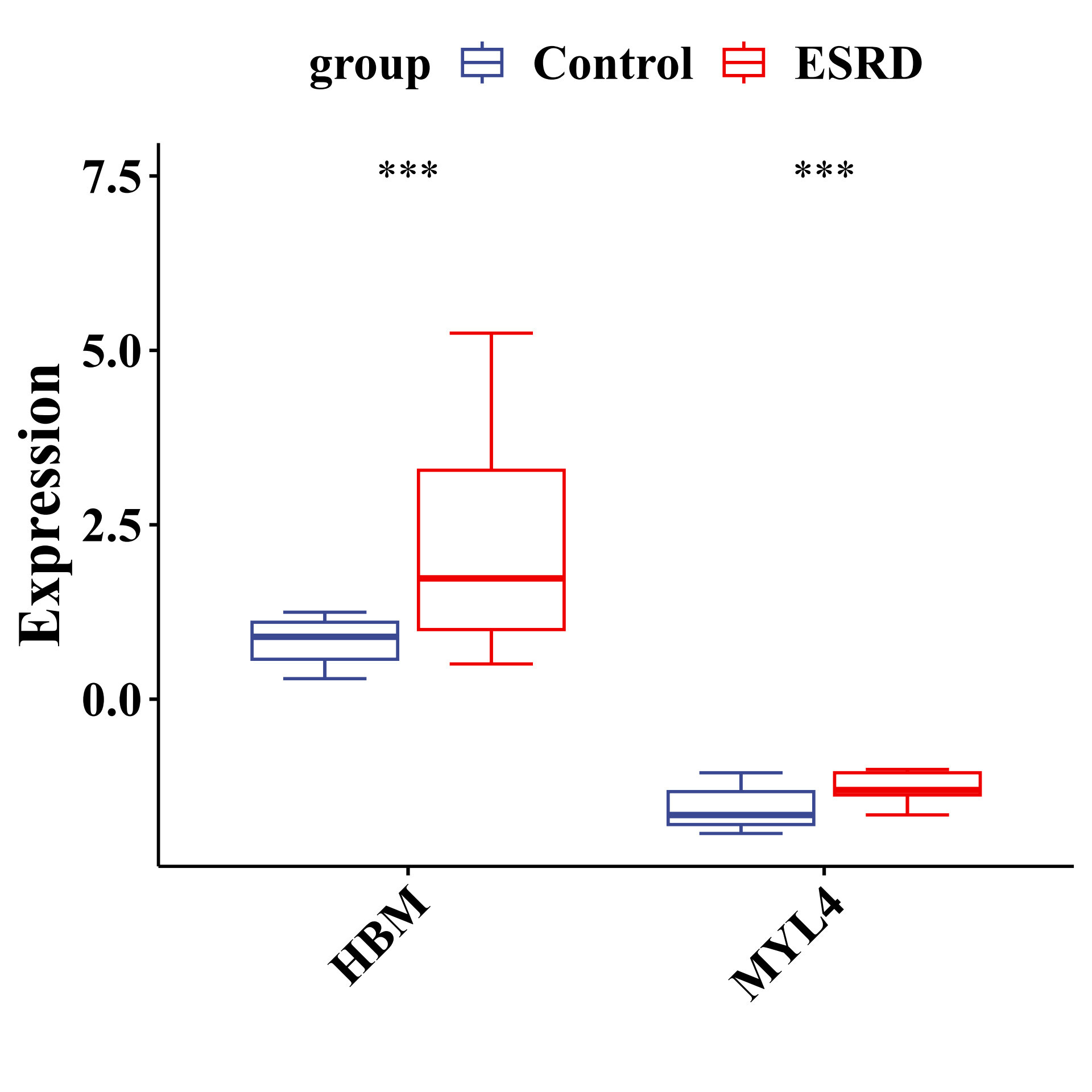

Supplement: _.zip [file IRNF_A_2519834_SM0592.zip › 图片终稿/Figure 4D.jpg]

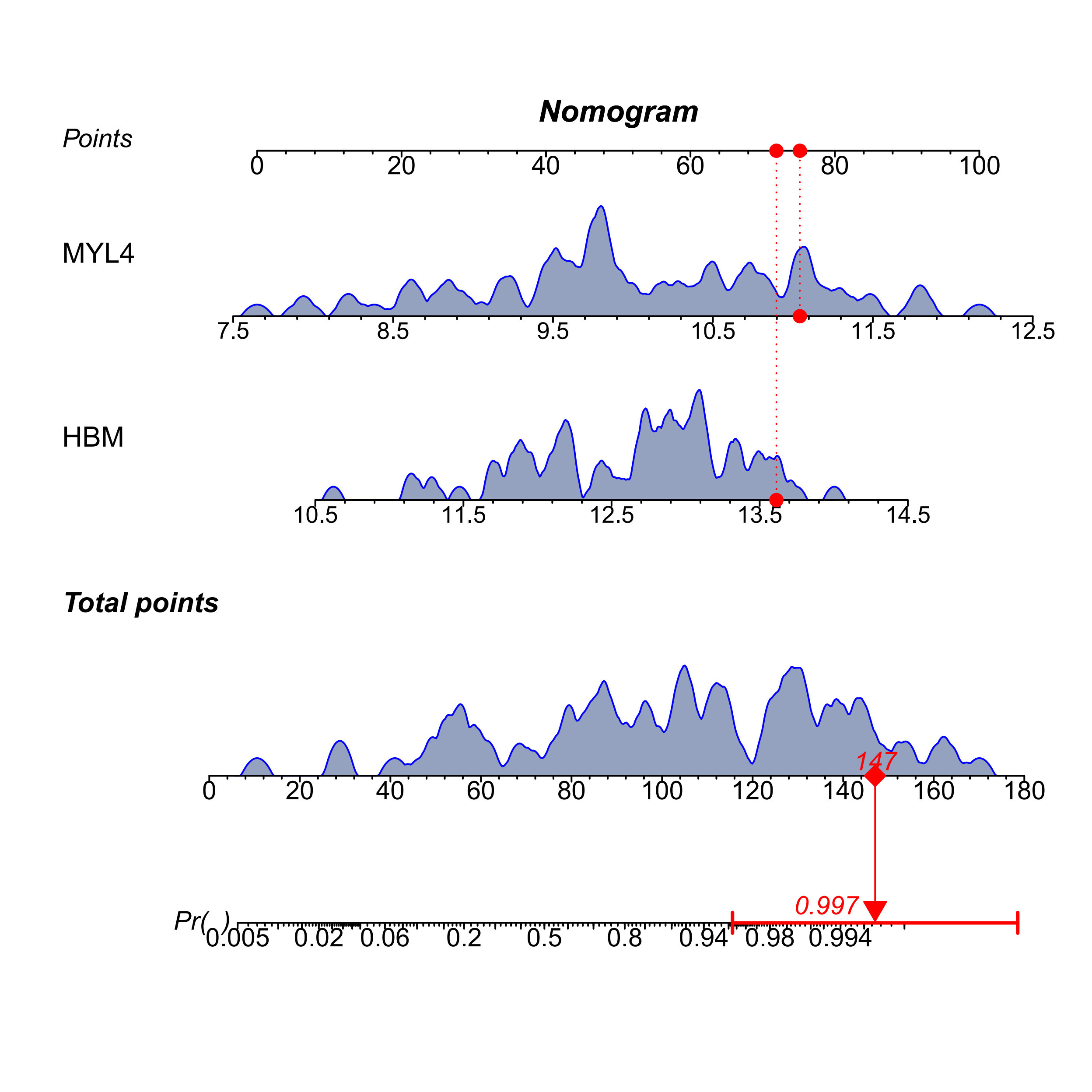

Supplement: _.zip [file IRNF_A_2519834_SM0592.zip › 图片终稿/Figure 4E.jpg]

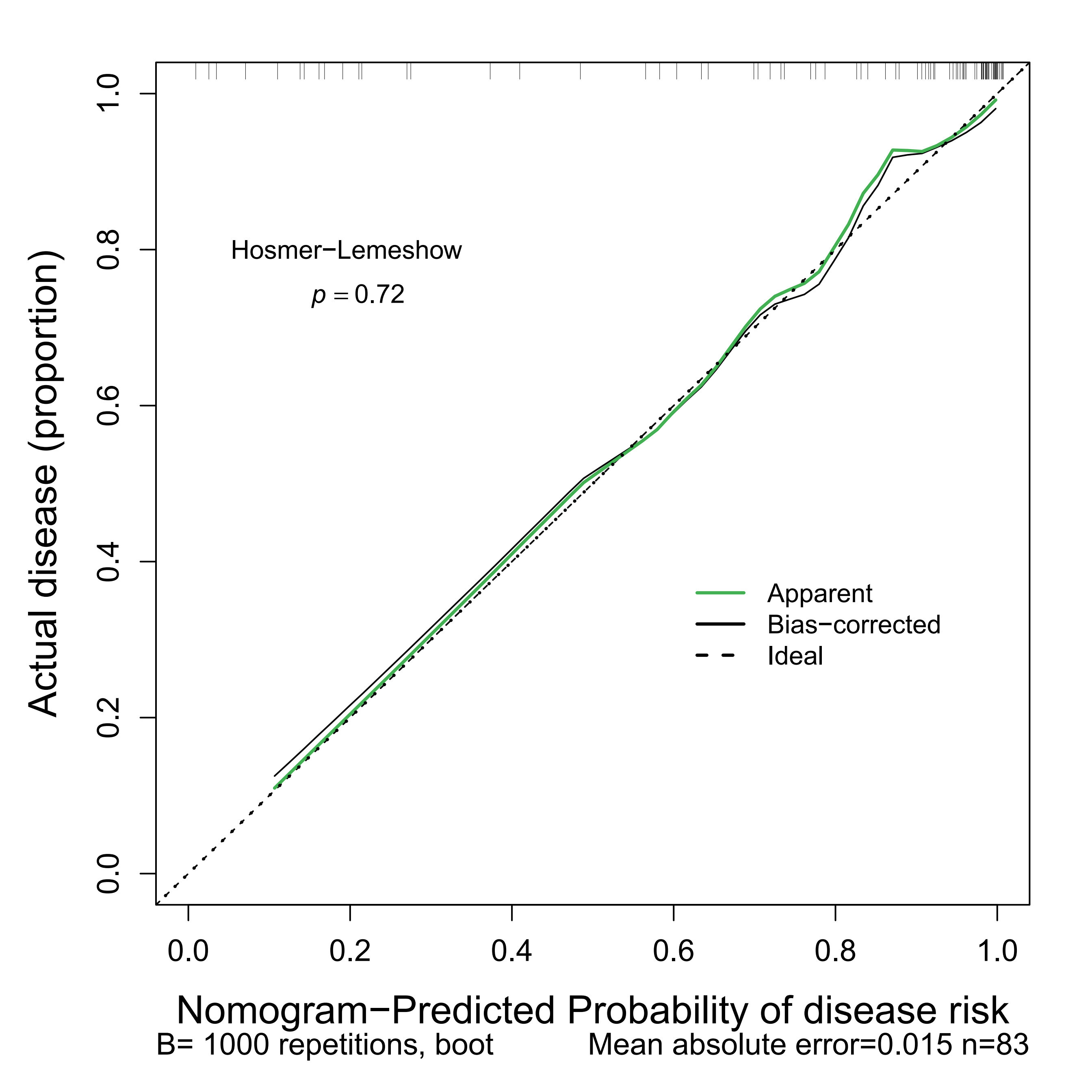

Supplement: _.zip [file IRNF_A_2519834_SM0592.zip › 图片终稿/Figure 4F.jpg]

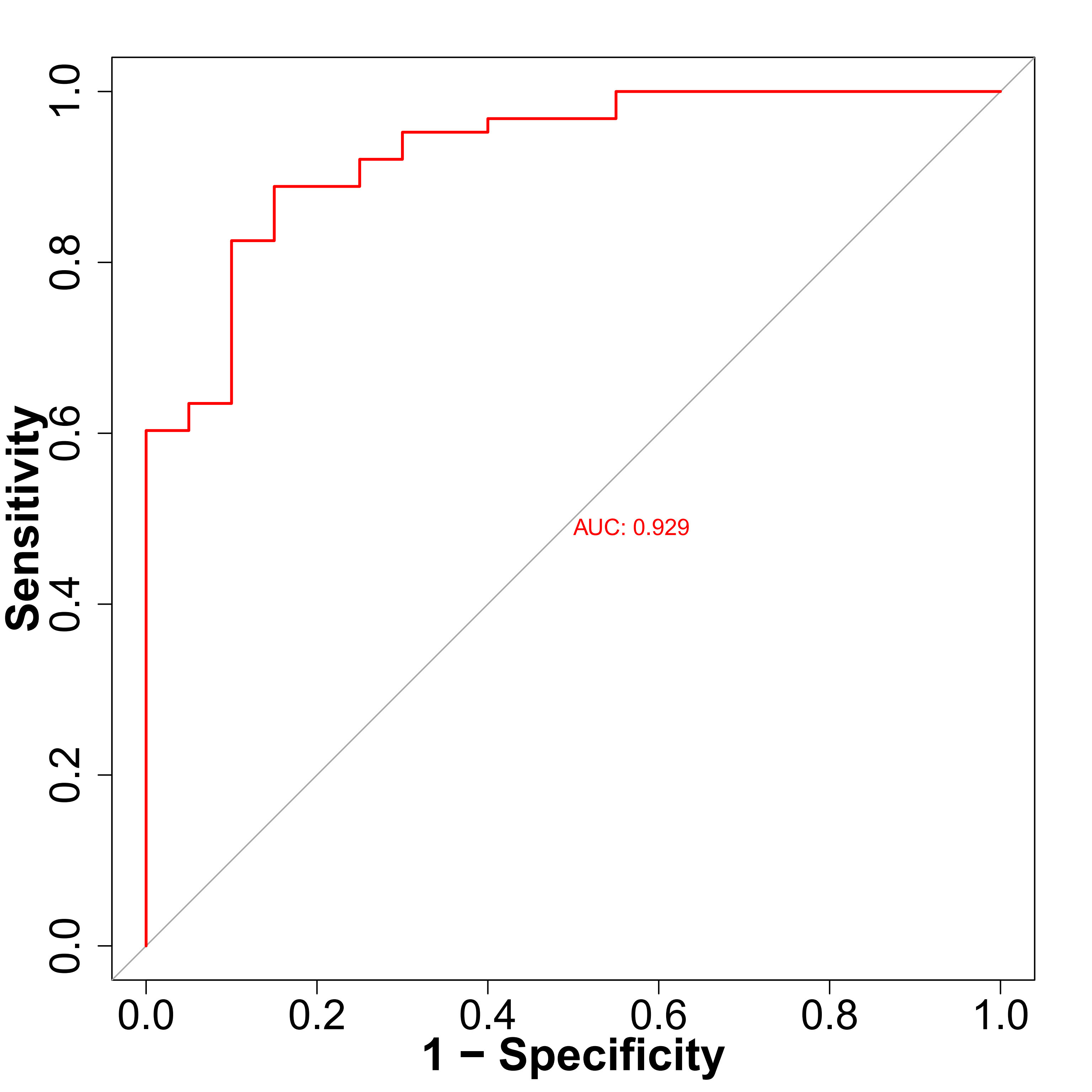

Supplement: _.zip [file IRNF_A_2519834_SM0592.zip › 图片终稿/Figure 4G.jpg]

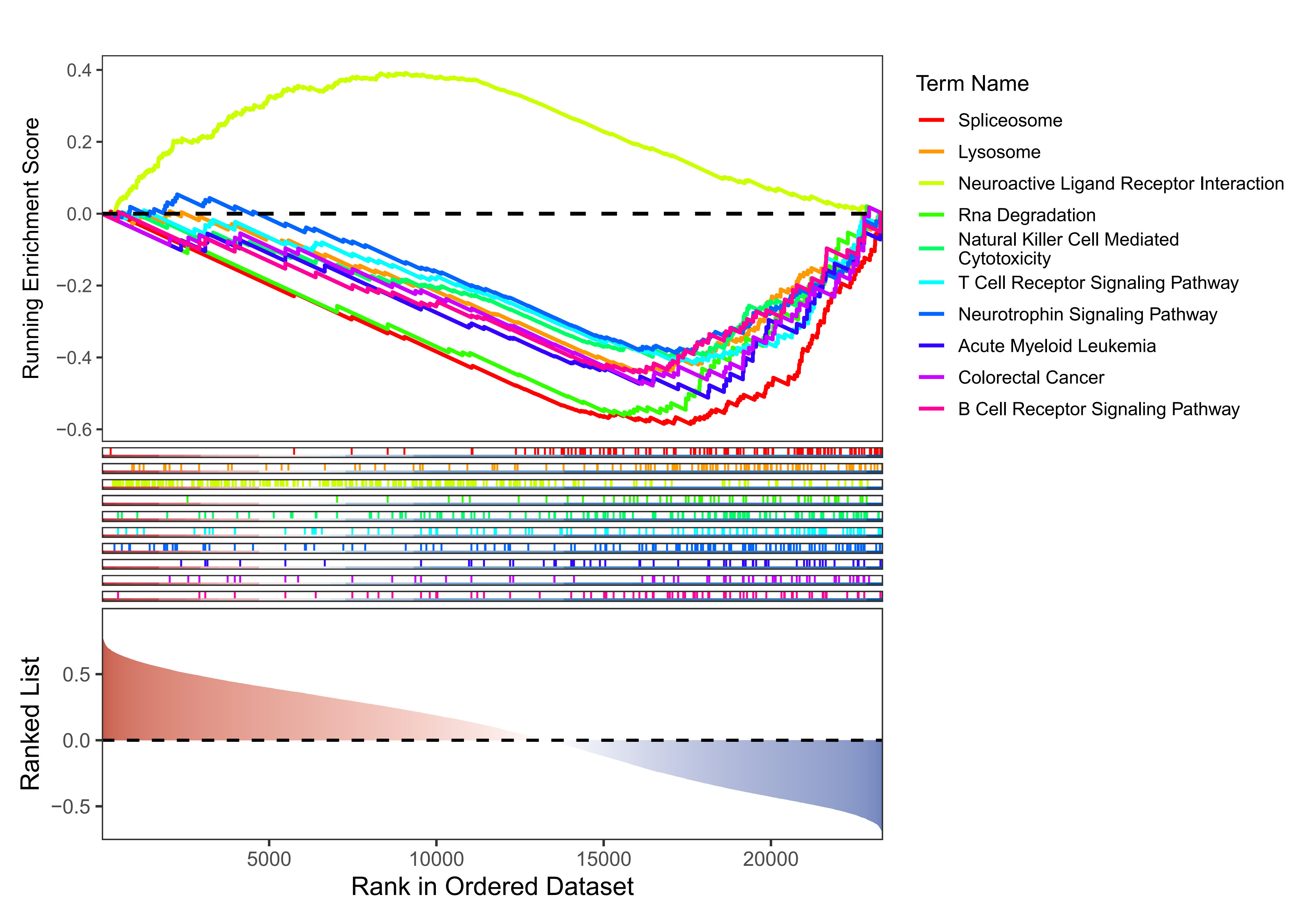

Supplement: _.zip [file IRNF_A_2519834_SM0592.zip › 图片终稿/Figure 5A.jpg]

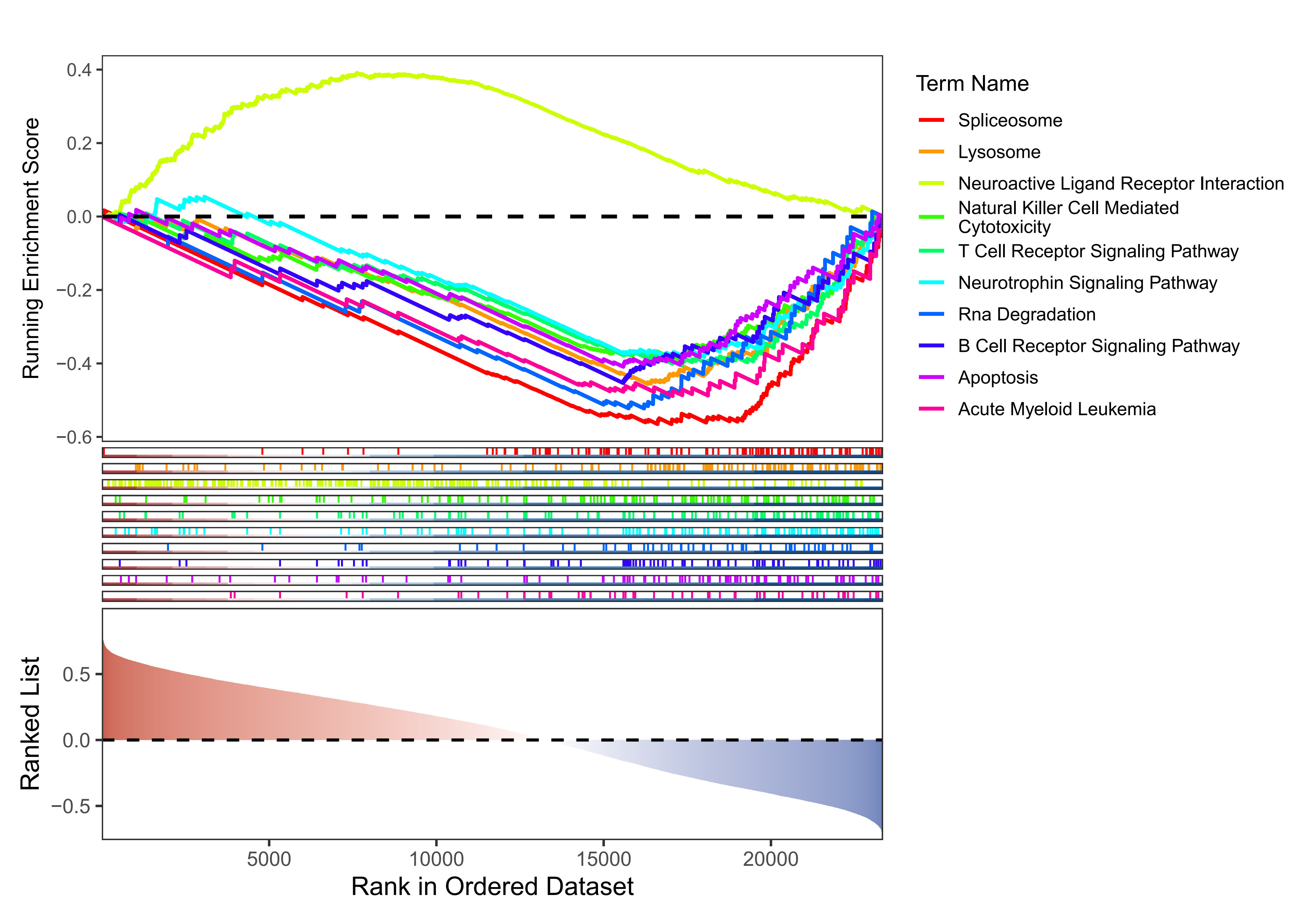

Supplement: _.zip [file IRNF_A_2519834_SM0592.zip › 图片终稿/Figure 5B.jpg]

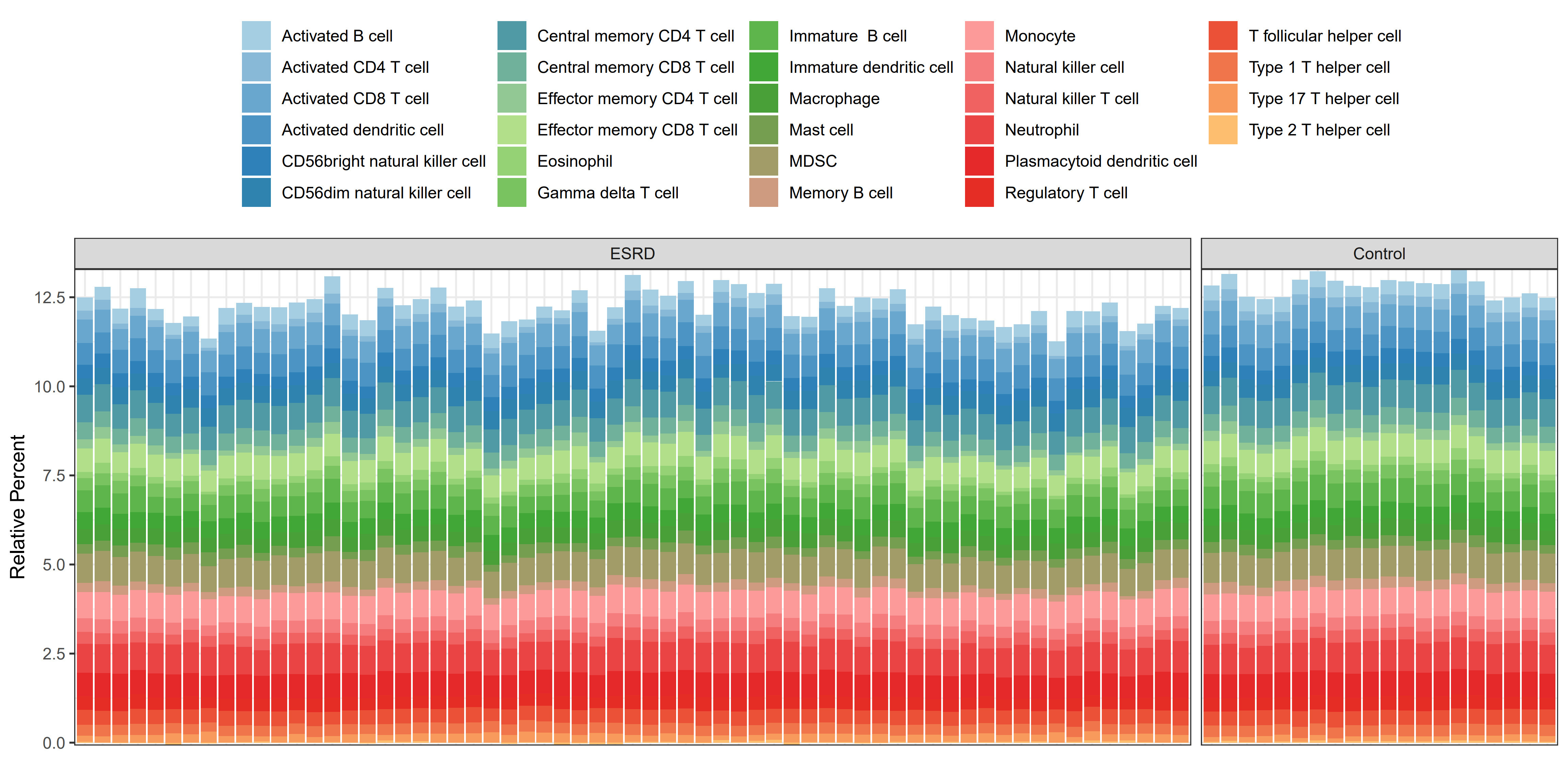

Supplement: _.zip [file IRNF_A_2519834_SM0592.zip › 图片终稿/Figure 5C.jpg]

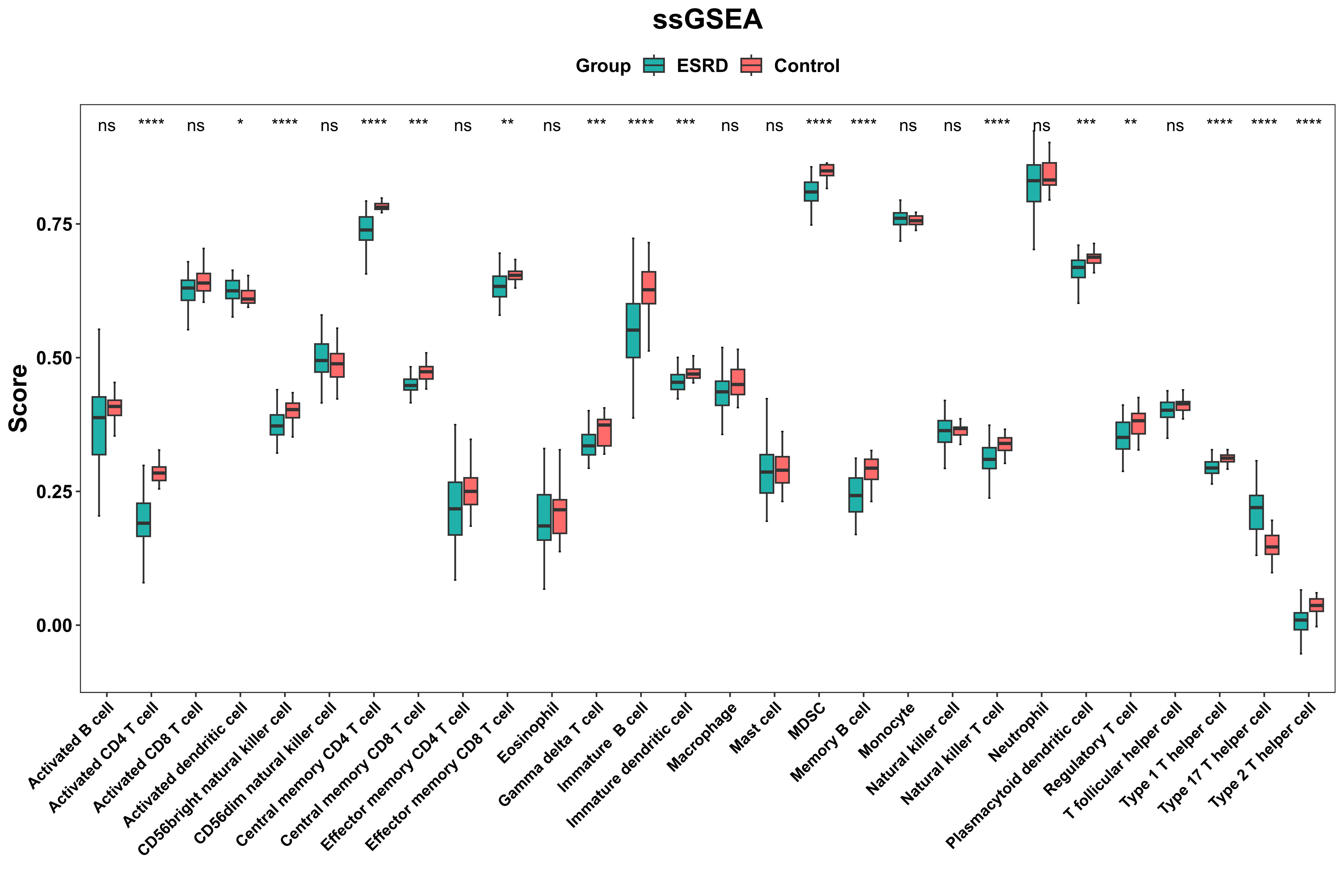

Supplement: _.zip [file IRNF_A_2519834_SM0592.zip › 图片终稿/Figure 5D.jpg]

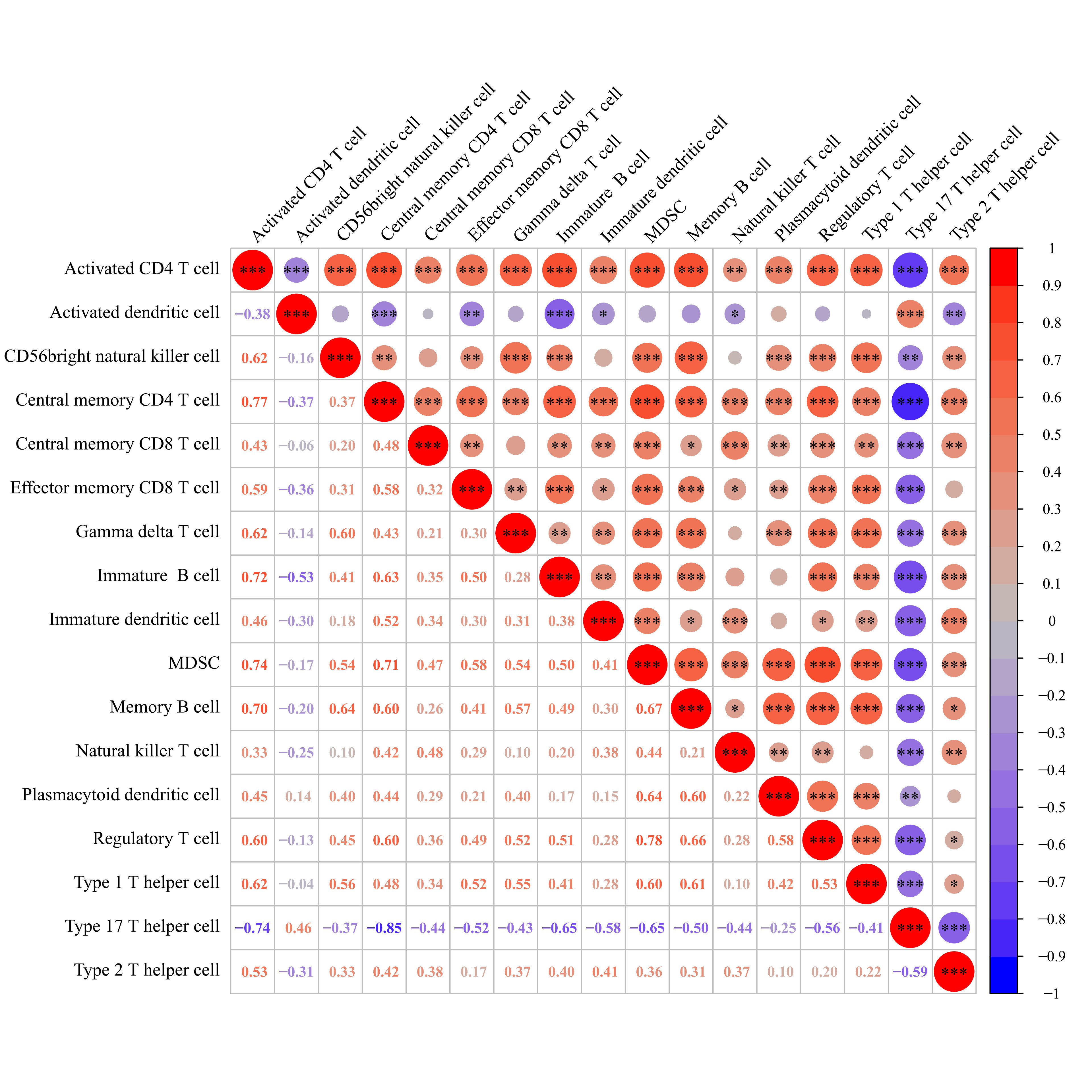

Supplement: _.zip [file IRNF_A_2519834_SM0592.zip › 图片终稿/Figure 5E.jpg]

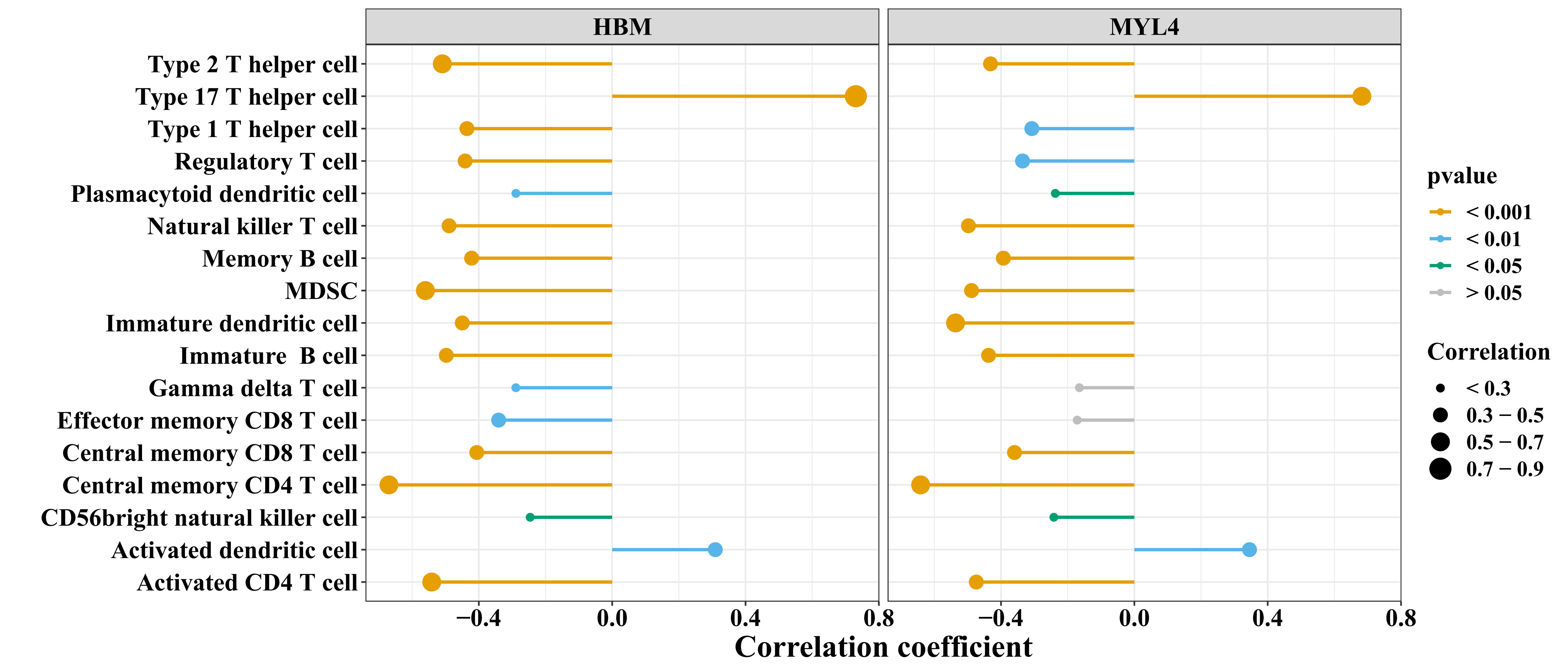

Supplement: _.zip [file IRNF_A_2519834_SM0592.zip › 图片终稿/Figure 5F.jpg]

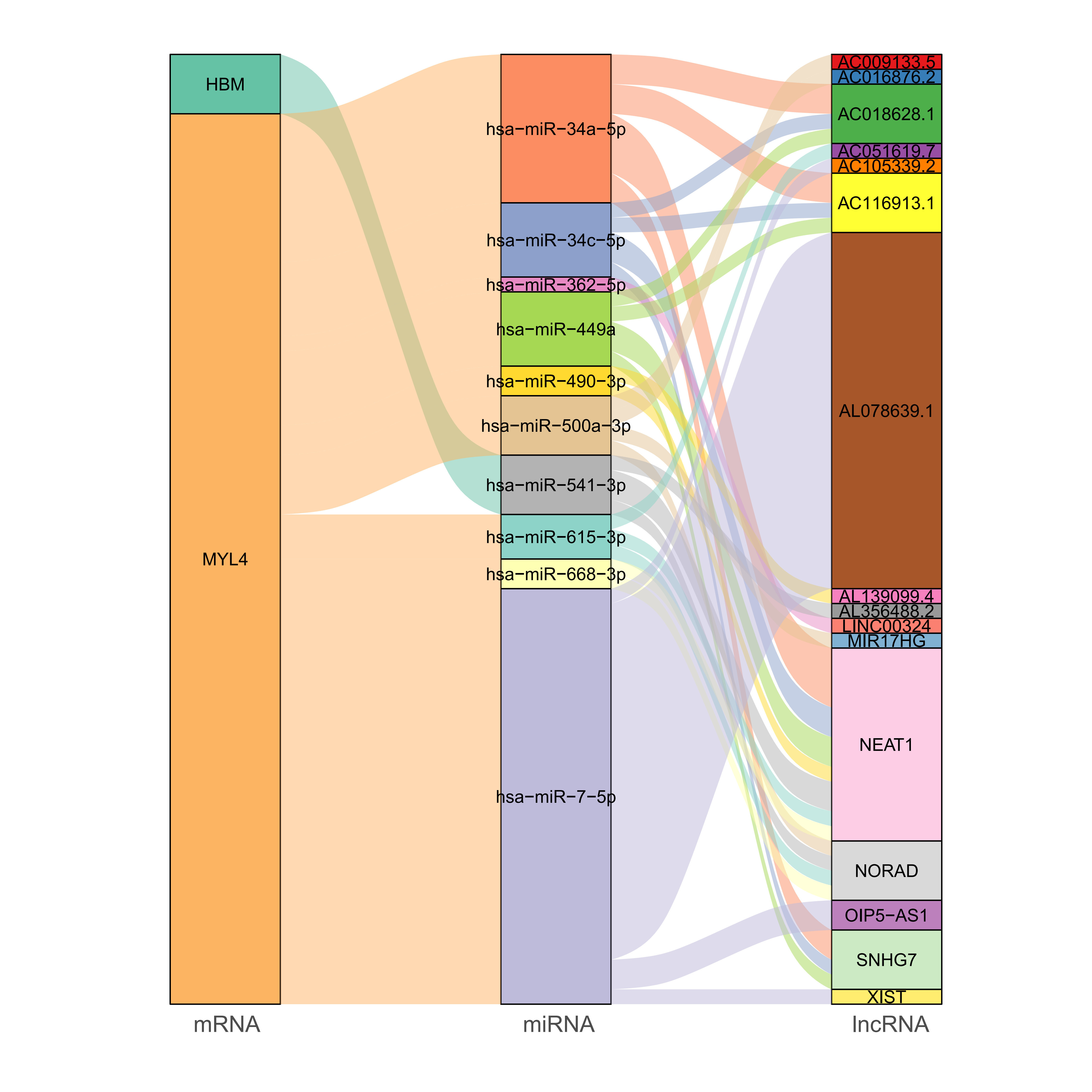

Supplement: _.zip [file IRNF_A_2519834_SM0592.zip › 图片终稿/Figure 6A.jpg]

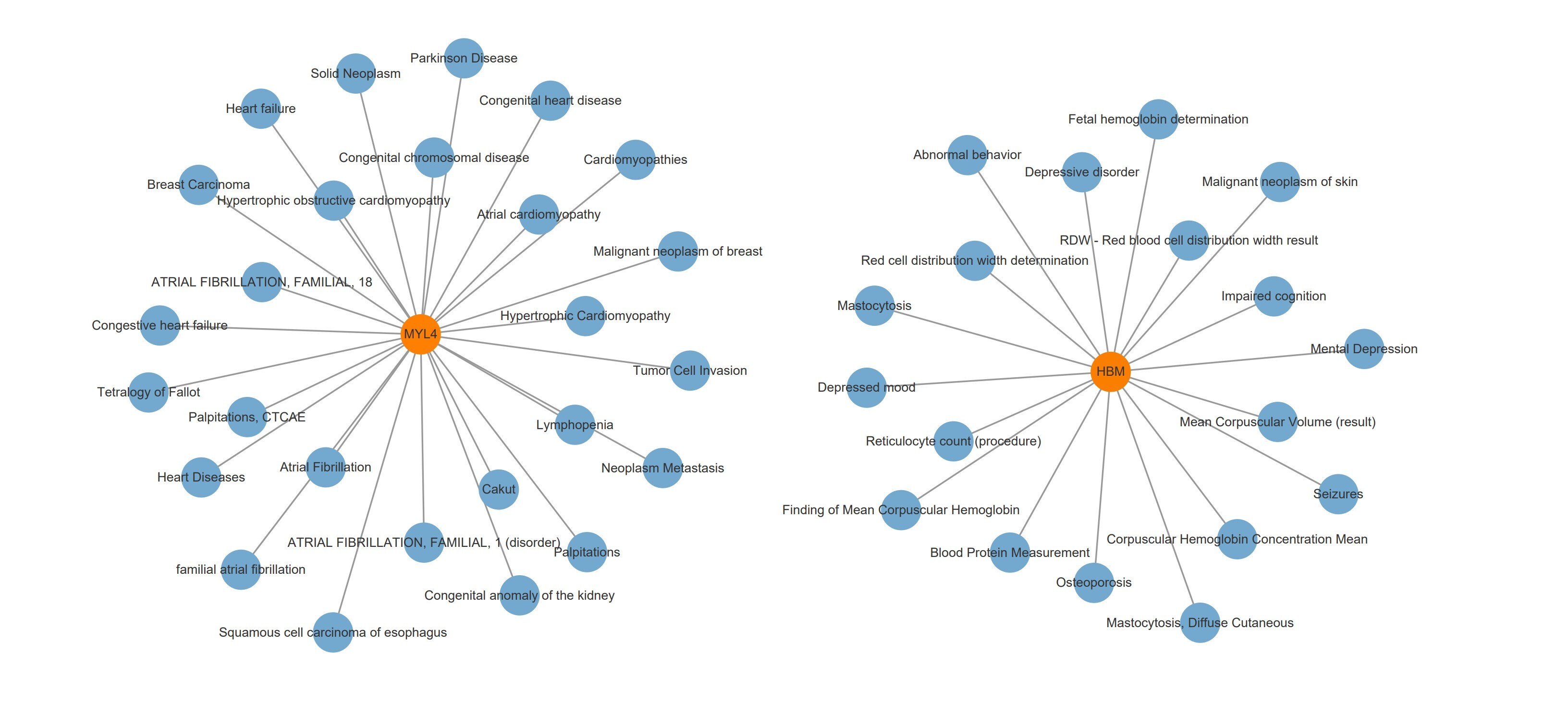

Supplement: _.zip [file IRNF_A_2519834_SM0592.zip › 图片终稿/Figure 6B.jpg]

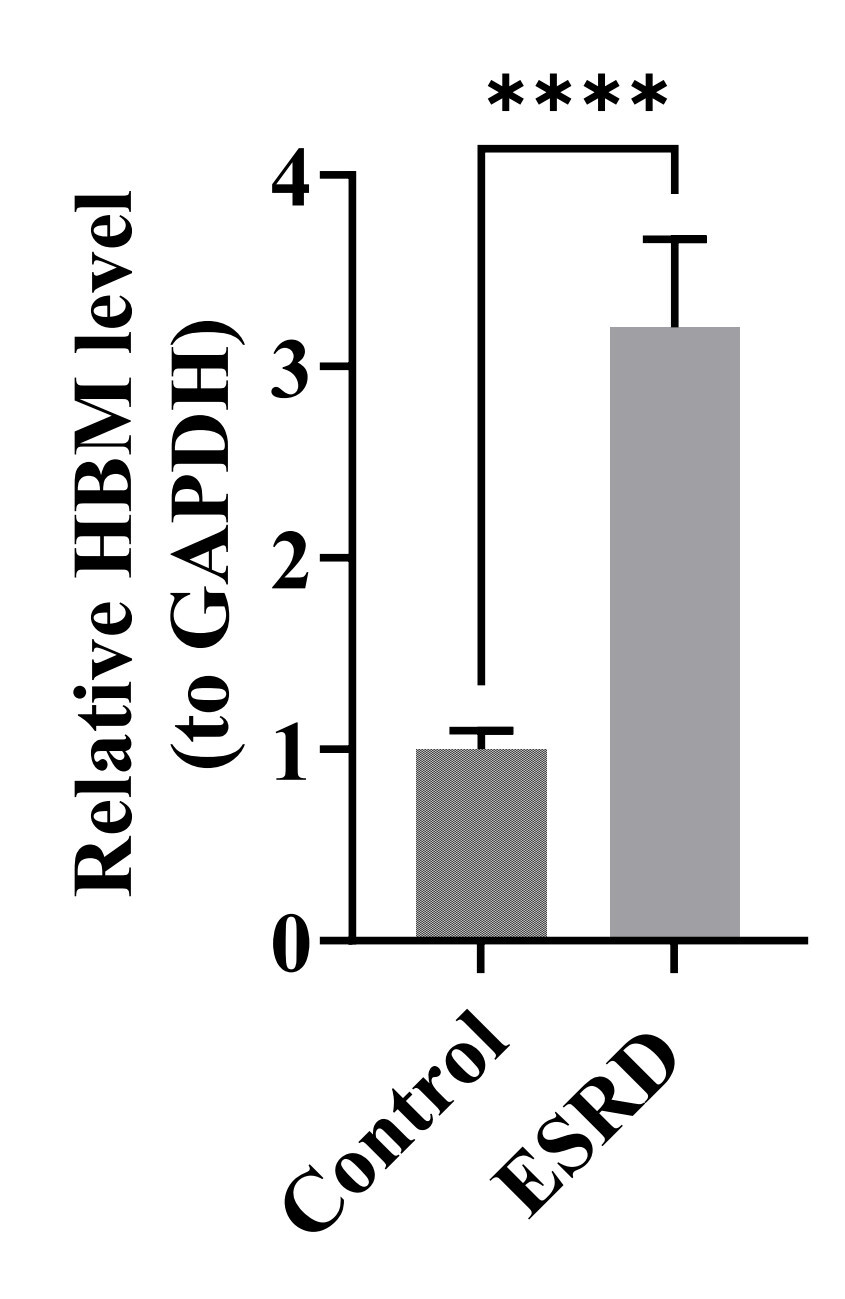

Supplement: _.zip [file IRNF_A_2519834_SM0592.zip › 图片终稿/Figure 7A.jpg]

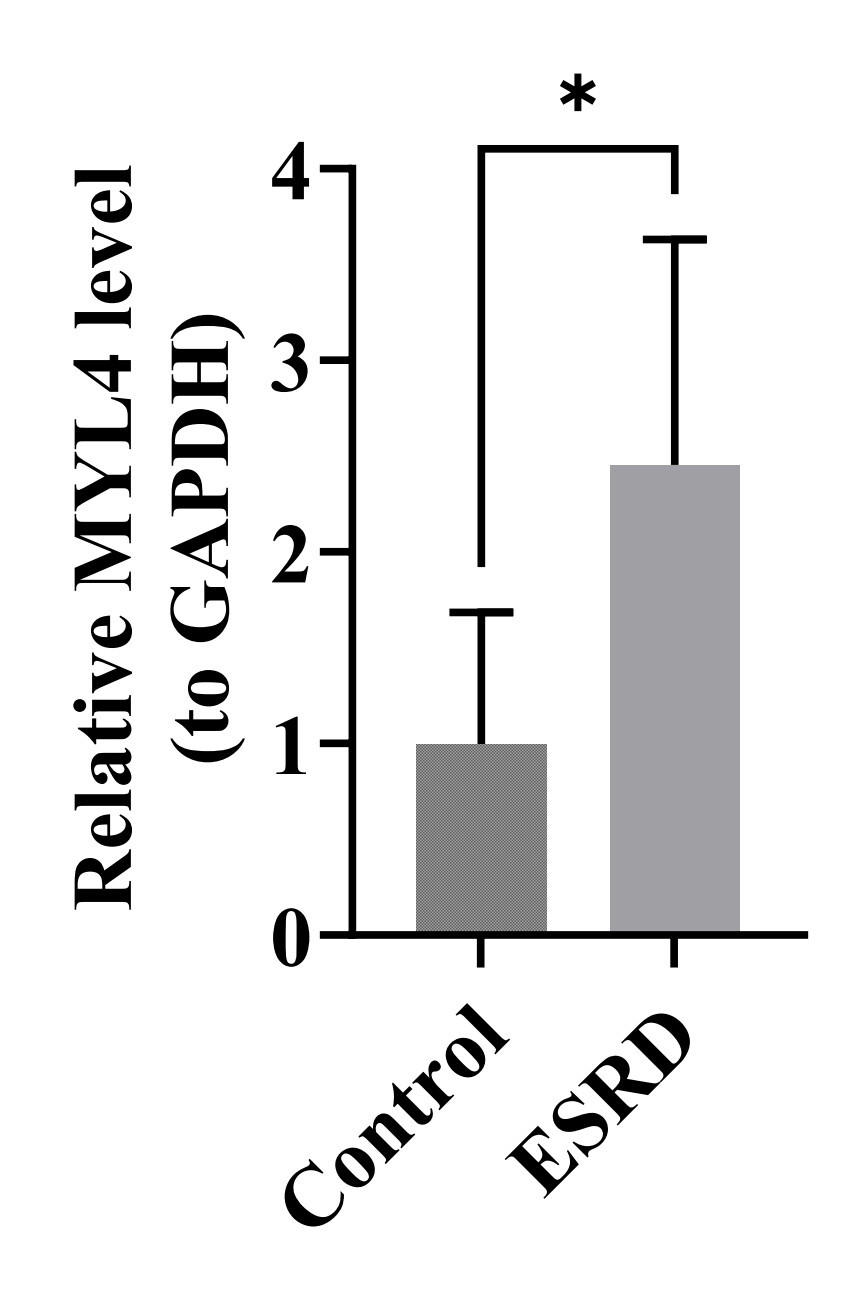

Supplement: _.zip [file IRNF_A_2519834_SM0592.zip › 图片终稿/Figure 7B.jpg]
