# Supplementary material for: Identification and validation of parthanatos-related genes in end-stage renal disease
Source: Ren Fail. 2025 Jul 6;47(1):2519834. doi: 10.1080/0886022X.2025.2519834 (PMC12231245; doi:10.1080/0886022X.2025.2519834)
Supplement: _.zip [file IRNF_A_2519834_SM0592.zip › 图片终稿/Table S1.docx]

Table S1 Information of primers

| **Primers** | **Sequences (5’-3’)** | |
| --- | --- | --- |
| HBM-F | ACGAGTTCACCGTGCAAATG | |
| HBM-R | ATTTTTCGGTCAGCACCACG | |
| MYL4-F | TGGGCTTTGTGTGAGGCTTA |  |
| MYL4-R | ACCTTCAATCTGGTCGGCAG |  |
| GAPDH-F | CGAAGGTGGAGTCAACGGATTT |  |
| GAPDH-R | ATGGGTGGAATCATATTGGAAC |  |
